# Deep learning model of fMRI connectivity predicts PTSD symptom trajectories in recent trauma survivors

**DOI:** 10.1016/j.neuroimage.2021.118242

**Published:** 2021-09

**Authors:** Shelly Sheynin, Lior Wolf, Ziv Ben-Zion, Jony Sheynin, Shira Reznik, Jackob Nimrod Keynan, Roee Admon, Arieh Shalev, Talma Hendler, Israel Liberzon

**Affiliations:** a School of Computer Science, Tel Aviv University, Tel-Aviv, Israel; b Sagol Brain Institute Tel-Aviv, Wohl Institute for Advanced Imaging, Tel Aviv Sourasky Medical Center, Tel-Aviv, Israel; c Sagol School of Neuroscience, Tel-Aviv University, Tel Aviv, Israel; d Department of Psychiatry and Behavioral Science, Stanford University School of Medicine, Stanford, USA; e Department of Psychiatry and Behavioral Science, Texas A&M University Health Science Center, TX, USA; f School of Psychological Sciences, University of Haifa, Haifa, Israel; g The Integrated Brain and Behavior Research Center (IBBRC), University of Haifa, Haifa, Israel; h Department of Psychiatry, New York University Grossman School of Medicine, New York, NY, USA; i School of Psychological Sciences, Faculty of Social Sciences, Tel-Aviv University, Tel-Aviv, Israel; j Sackler Faculty of Medicine, Tel-Aviv University, Tel-Aviv, Israel

**Keywords:** fMRI, Deep learning, Attention mechanism, End-to-end neural network, PTSD symptom clusters

## Abstract

Early intervention following exposure to a traumatic life event could change the clinical path from the development of post traumatic stress disorder (PTSD) to recovery, hence the interest in early detection and underlying biological mechanisms involved in the development of post traumatic sequelae. We introduce a novel end-to-end neural network that employs resting-state and task-based functional MRI (fMRI) datasets, obtained one month after trauma exposure, to predict PTSD symptoms at one-, six- and fourteen-months after the exposure. FMRI data, as well as PTSD status and symptoms, were collected from adults at risk for PTSD development, after admission to emergency room following a traumatic event. Our computational method utilized a per-region encoder to extract brain regions embedding, which were subsequently updated by applying the algorithmic technique of pairwise attention. The affinities obtained between each pair of regions were combined to create a pairwise co-activation map used to perform multi-label classification. The results demonstrate that the novel method’s performance in predicting PTSD symptoms, in a prospective manner, outperforms previous analytical techniques reported in the fMRI literature, all trained on the same dataset. We further show a high predictive ability for predicting PTSD symptom clusters and PTSD persistence. To the best of our knowledge, this is the first deep learning method applied on fMRI data with respect to prospective clinical outcomes, to predict PTSD status, severity and symptom clusters. Future work could further delineate the mechanisms that underlie such a prediction, and potentially improve single patient characterization.

## Highlights

• We propose a novel end-to-end neural network that employs resting-state and task-based functional MRI (fMRI) datasets, obtained one month after trauma exposure, to predict PTSD one, six and 14-months after the exposure.
• The method utilizes connectivity maps extracted from pairs of brain regions which are subsequently updated by applying the algorithmic technique of pairwise attention.
• The proposed deep learning method predicts PTSD status, PTSD symptom clusters and survival analysis within the prospective design. We demonstrate a significant improvement in performance on all the datasets and experiments in comparison to other relevant analytical techniques.
• Pairwise association analysis reveals several significant functional connectivity patterns, in line with previous PTSD neuroimaging literature.

## 1 Introduction

Post-traumatic stress disorder (PTSD) is a common psychiatric disorder with significant clinical and public health impact, due to its high prevalence, chronicity, associated functional impairment and frequent comorbidities (Kessler, 2000, Shalev, Liberzon, Marmar, 2017). While most trauma-exposed survivors who develop initial PTSD symptoms exhibit rapid remission (about 56%) or show delayed/partial remission (about 27%) over fourteen months, a subset of about 17% do not remit and suffer from chronic PTSD (Galatzer-Levy et al., 2013). The development of PTSD in a subgroup of survivors, and the tenacity of the protracted disorder, suggest a long-lasting trauma induced neuro-behavioral alteration (Pitman et al., 2012). Longitudinal studies examining multi-modal dimensions of the response to trauma (e.g., symptoms, cognitive functions, brain structure and functioning) are optimally suited to detect the underlying neuro-behavioral moderators of non remitting PTSD (Ben-Zion, Artzi, Niry, Keynan, Zeevi, et al., 2019, Ben-Zion, Fine, Keynan, Admon, Green, Halevi, Fonzo, Achituv, et al., 2018, Pitman, Rasmusson, Koenen, Shin, Orr, Gilbertson, Milad, Liberzon, 2012, Shalev, Liberzon, Marmar, 2017).

Converging neuroimaging studies have suggested abnormalities in brain regions involved in emotional processing in individuals with PTSD (Etkin, Wager, 2007, Shin, Liberzon, 2010). These include emotional reactivity and salience processing abnormalities in the amygdala, insula, and dorsal anterior cingulate cortex (ACC), and emotion-regulation and contextual processing abnormalities in medial and lateral prefrontal cortices, and ventral ACC (Etkin, Büchel, Gross, 2015, Pitman, Rasmusson, Koenen, Shin, Orr, Gilbertson, Milad, Liberzon, 2012, Rabinak, Angstadt, Welsh, Kennedy, Lyubkin, Martis, Phan, 2011, Shalev, Liberzon, Marmar, 2017, Sripada, King, Garfinkel, Wang, Sripada, Welsh, Liberzon, 2012, Sripada, King, Welsh, Garfinkel, Wang, Sripada, Liberzon, 2012).

Nevertheless, these findings come mainly from cross-sectional studies comparing a group of PTSD patients to a group of trauma-exposed individuals who did not develop PTSD. Moreover, the dynamics in clinical symptoms observed through the first year after trauma points to associated changes over time in underlying neural processing(Bryant, Nickerson, Creamer, O’Donnell, Forbes, Galatzer-Levy, McFarlane, Silove, 2015, Galatzer-Levy, Ankri, Freedman, Israeli-Shalev, Roitman, Gilad, Shalev, 2013, Shalev, Gevonden, Ratanatharathorn, Laska, Van Der Mei, Qi, Lowe, Lai, Bryant, Delahanty, et al., 2019). To date, in studies attempting classification of PTSD, prospective data driven investigations have been scarce, and mainly relied on hypotheses regarding known brain abnormalities in PTSD. Previous large scale studies have mostly utilized resting state fMRI (rs-fMRI) or structural MRI (for example; (Brown, LaBar, Haswell, Gold, McCarthy, Morey, 2014, Rabinak, Angstadt, Welsh, Kennedy, Lyubkin, Martis, Phan, 2011)), yet studies of whole brain data-driven analysis both at rest and during different tasks, are still needed (Ben-Zion, Zeevi, Keynan, Admon, Kozlovski, Sharon, Halpern, Liberzon, Shalev, Benjamini, et al., 2020, DiGangi, Tadayyon, Fitzgerald, et al., 2016, King, Block, Sripada, Rauch, Giardino, Favorite, Angstadt, Kessler, Welsh, Liberzon, 2016, Wang, Zhu, et al., 2020). Investigating brain connectivity during task in addition to rest could enhance the validity of finding with respect to real life mental processing.

In addition, to date, work on PTSD classification has largely overlooked the dynamics of evolving PTSD symptom trajectories and considered single time-point outcomes. Some longitudinal studies (Kendrick, Baker, Hill, Beckett, Coupland, Kellezi, Joseph, Barnes, Sleney, Christie, et al., 2018, Mason, Wardrope, Turpin, Rowlands, 2002, Qi, Ratanatharathorn, Gevonden, Bryant, Delahanty, Matsuoka, Olff, deRoon Cassini, Schnyder, Seedat, et al., 2018, Richmond, Ruzek, Ackerson, Wiebe, Winston, Kassam-Adams, 2011, Scher, Suvak, Resick, 2017, Shalev, Gevonden, Ratanatharathorn, Laska, Van Der Mei, Qi, Lowe, Lai, Bryant, Delahanty, et al., 2019) yielded both group and individual level PTSD risk prediction based on early behavioral and psychological measures, yet, the question of underlying neurobehavioral mechanisms remains open. Not surprisingly, meta-analyses (Brewin, Andrews, Valentine, 2000, Ozer, Best, Lipsey, Weiss, 2003) and systematic reviews (Brewin, 2005, Brewin, Andrews, Valentine, 2000, Heron-Delaney, Kenardy, Charlton, Matsuoka, 2013, Ozer, Best, Lipsey, Weiss, 2003) have focused on group-level risk indicators without a clear path to clinical implementation at the individual level (Heron-Delaney et al., 2013). All of these gaps, warrant the current prospective investigation of underlying brain mechanisms using our novel end-to-end neural network on resting state and task fMRI data obtained closely following trauma exposure.

Another challenge in examining the trajectory of PTSD following trauma is the heterogeneity of clinical symptoms. According to DSM-5, PTSD consists of four symptom clusters: Intrusion (Criteria B), Avoidance (Criteria C), Negative alterations in mood and cognition (Criteria D), and Hyperaousal (Criteria E); raising the possibility that different symptom clusters may reflect different mechanisms/processes involved. It is therefore of the utmost importance to further consider, not only PTSD diagnosis but also the specific symptom cluster or constellation. However, the majority of studies finding qualitatively distinct profiles among diagnosed individuals have not been utilized to predict the four symptom clusters of PTSD. In this work, we fill this gap by training our deep learning model to identify distinct, symptom-based clusters of PTSD according to DSM-5 criteria.

Machine learning (ML) approaches are increasingly utilized to overcome the problem of characterization, prediction, and treatment selection for individuals suffering from a variety of psychiatric disorders (Schultebraucks and Galatzer-Levy, 2019). For example, Galatzer-Levy et al. (2017) employed a support vector machine (SVM) for prediction of PTSD trajectories of trauma survivors and Rosellini et al. (2018) used an ensemble of multiple classifiers, including random forests (Breiman, 2001), SVM, and regularized regression, to develop a risk score for earthquake survivors. Recently, a growing number of studies have applied ML methodology on neuroimaging data to predict and characterize a variety of psychiatric disorders (Bleich-Cohen, Jamshy, Sharon, Weizman, Intrator, Poyurovsky, Hendler, 2014, Haller, Lovblad, Giannakopoulos, Van De Ville, 2014, Koutsouleris, Kambeitz, 2016, Liu, Guo, Yu, Gao, Gao, Xue, Du, Zhang, Tan, Liu, et al., 2012, Mikolas, Melicher, Skoch, Matejka, Slovakova, Bakstein, Hajek, Spaniel, 2016, Mourao-Miranda, Reinders, Rocha-Rego, Lappin, Rondina, Morgan, Morgan, Fearon, Jones, Doody, et al., 2012, van der Ploeg, Nieboer, Steyerberg, 2016, Rive, Redlich, Schmaal, Marquand, Dannlowski, Grotegerd, Veltman, Schene, Ruhé, 2016, Zeng, Shen, Liu, Wang, Li, Fang, Zhou, Li, Hu, 2012), including PTSD (Galatzer-Levy, Ma, Statnikov, et al., 2017, Gong, Li, Du, Pettersson-Yeo, Crossley, Yang, Li, Huang, Mechelli, 2014, Gradus, King, Galatzer-Levy, Street, 2017, Jin, Jia, Lanka, Rangaprakash, Li, Liu, Hu, Deshpande, 2017, Karstoft, Galatzer-Levy, Statnikov, Li, Shalev, et al., 2015, Liu, Xie, Wang, Guo, Fouche, Long, Wang, Chen, Li, Duan, et al., 2015, Omurca, Ekinci, 2015, Saxe, Ma, Ren, Aliferis, 2017, Schultebraucks, Galatzer-Levy, 2019). To date, however, no studies have applied a single ML method on both resting-state and task-based neuroimaging data to classify PTSD diagnosis simultaneously at three different time-points, using brain data only from the first time-point.

With the advent of deep learning (DL) methods, a variety of neural network approaches have been applied for fMRI classification, including autoencoders (Patel et al., 2016), Recurrent Neural Networks (Dakka et al., 2017) and Convolutional Neural Networks (CNNs) (Bengs, Gessert, Schlaefer, 2019, Khosla, Jamison, Kuceyeski, Sabuncu, 2018, Mao, Su, Xu, Wang, Huang, Yue, et al., 2019, Riaz, Asad, Arif, Alonso, Dima, Corr, Slabaugh, 2018, Zou, Zheng, et al., 2017). Several recent reports (Bengs, Gessert, Schlaefer, 2019, Mao, Su, Xu, Wang, Huang, Yue, et al., 2019) treated the fMRI data as a 4D volume and used a 4D CNN network to learn spatial and temporal features simultaneously. Connectivity fingerprints, created as multi-channel tensors recording the coupling of each voxel to distinct target Regions of Interest (ROIs), were used as input to a CNN (Khosla et al., 2018). Li et al. (2019) used an inductive graph neural network (GNN) on top of elaborate summary vectors to obtain ASD (Autism Spectrum Disorders) classification. Other methods used summary statistics per region of interest to perform classification. For example, Riaz et al. (2018) used the pooled activations across multiple regions to compute features and obtain pairwise similarities in activations, which are used for classification. However, none of these deep learning methods have been applied in the field of PTSD to date.

In this work, we used clinical data and neural indices, collected shortly after trauma to predict the course of PTSD severity, including distinct clusters of PTSD-related symptom/variables, during the first year after trauma exposure (Ben-Zion et al., 2019b). We additionally applied survival analysis within our prospective design, to predict the persistence of PTSD. Finally, statistical tests were applied to identify pairwise correlations in our PTSD prediction model which discriminate between PTSD patients and controls. The neural data included fMRI scans collected within one-month after trauma during: resting state, emotional reactivity task (Hariri et al., 2000), and Safe or Risky Domino Choice (SRDC) task (Ben-Zion, Shany, Admon, Keynan, Avisdris, Balter, Shalev, Liberzon, Hendler, Kahn, Yeshurun, Rotshtein, Fried, Ben-Bashat, Hendler, 2002). The clinical diagnosis was obtained using the Clinician-Administered PTSD Scale (CAPS) instrument (Weathers et al., 2018), at one-, six- and fourteen-months post-trauma.

Our novel analytical methodology utilized connectivity (correlation) maps extracted from pairs of brain regions, in a whole-brain analysis. These connectivities were obtained using a novel end-to-end neural network by first computing embedding for every brain region and then further applying an attention mechanism (Vaswani et al., 2017), which allowed to focus on the most informative features of the input. Using the attention mechanism, we aggregated together the embeddings that demonstrated the highest similarities. These combined embeddings were subsequently used to produce whole-brain functional connectivity maps for our classifier.

To benchmark our predictive model, we compared the model with previously used ML and DL approaches by training all the techniques from scratch on the same dataset, showing a significant improvement on all the tasks, both in classification accuracy, AUC (Area Under the Curve) and average precision.

To our knowledge, this is the first report of deep learning method used to predict PTSD diagnosis and symptom clusters (based on DSM-5), in a prospective design, using neural data obtained one-month following trauma. While we considered rs-fMRI to be the most common modality used in this type of analysis (Sripada, King, Garfinkel, Wang, Sripada, Welsh, Liberzon, 2012, Yuan, Phillips, Wong, Zotev, Misaki, Wurfel, Krueger, Feldner, Bodurka, 2018), we reproduced the high discriminatory performance of our method by also employing task-based fMRI. Thus, the predictive power of our method as well as its superiority over the algorithmic baselines, was demonstrated multiple times.

## 2 Methods

### 2.1 Participants

**Fig. 1:**
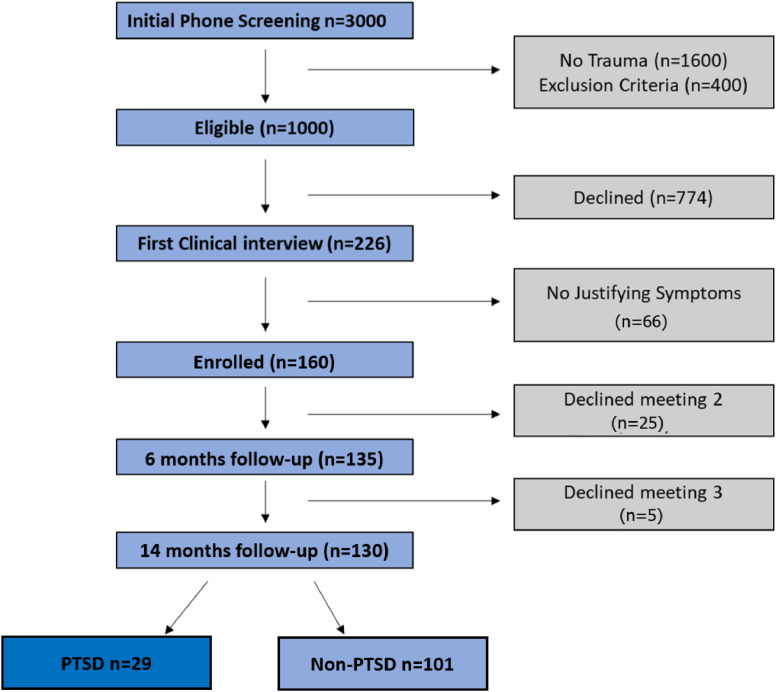
Consort diagram.

One hundred seventy-one adults (87 women, mean age 34.22 years, range 1865 years) who were admitted to a general hospitals emergency department after a traumatic event underwent clinical assessments and fMRI scans, at one-, six- and fourteen-months following trauma (T1, T2, and T3, respectively). Eleven participants were excluded from the analysis at T1, due to partial or low-quality data, resulting in a final dataset of *n*=160 individuals with valid fMRI and clinical data, obtained one-month after trauma (T1) (see ”Enrolled” in Fig. 1). Participants PTSD status and severity were determined by the CAPS) (Blake, Weathers, et al., 1995, Weathers, Bovin, Lee, Sloan, Schnurr, Kaloupek, Keane, Marx, 2018), a structured clinical interview corresponding to DSM-based PTSD criteria as determined by the dimensions of frequency, intensity, and severity of symptoms. The results shown by Stein et al. (2014) argue for a broad definition of PTSD defined by any one of the different systems to capture all clinically significant cases of PTSD in future studies. Thus, based on the recommendations to use a ”broad diagnostic approach”, individuals were included if they met PTSD diagnosis by either: (i) DSM-IV, (ii) DSM-5, or (iii) CAPS-IV total score of 40 or greater.

Out of the *n*=160 enrolled at T1, *n*=135 and *n*=130 completed clinical follow-up assessments at T2 and T3 (see Fig. 1). Twenty-five participants were lost to follow-up between T1 to T2, and an additional five participants were lost to follow-up between T2 and T3. While loss to follow-up in longitudinal studies is a well-known, often unresolved challenge, in this study it was relatively low. Importantly, no significant differences in age, gender, or initial symptom severity (CAPS total scores) were found between participants who completed the study and those who dropped out between T1 to T2 or between T2 to T3 (*p*>0.05 for all comparisons). Another challenge is imbalance label distribution, i.e., class imbalance: 72% of the subjects met criteria for PTSD diagnosis in T1, 29% in T2 and 23% in T3. We address the imbalance in Section 2.5.2. In Table 6 we present: (a) The number of participants, at each time-point, including their PTSD status in the test and training set (b) The number of participants, in each time-point, that meet the criteria for each symptom cluster in the test and training set as described in Section 2.5.5. Is is important to note that our model consists of training data only from the first time-point, and labels (PTSD/symptom cluster diagnosis) from all the three time-points.

**Table 1:** The number of participants in each time-point for a typical train/test split. The assignment between train and test is random at the patient level, and each patient may or may not participate in later on time-points, leading to variability between splits.

	Training			Test				Training			Test		
	T1	T2	T3	T1	T2	T3		T1	T2	T3	T1	T2	T3
Total	130	108	107	30	27	23	Total	130	108	107	30	27	23
PTSD	92	30	23	23	9	6	B	105	55	35	23	13	9
							C	97	47	32	22	14	8
							D	90	48	20	25	9	8
							E	96	55	43	22	15	9
(a)							(b)						

### 2.2 fMRI tasks

The data were collected and analyzed separately for patients performing three different fMRI paradigms: *(i) Resting-state fMRI (rs-fMRI) scan*. A 10 min rs-fMRI scan in which participants were instructed to keep their eyes open, focusing on a fixation cross. *(ii) Emotional Reactivity Task (**Hariri et al., 2000*) Here, participants were asked to select the face/shape that matched target face/shape, as accurately and quickly as possible. The task included four blocks of shapes, and four blocks of emotional faces (angry, fearful, surprised and neutral faces). *(iii) Safe or Risky Domino Choice task (SRDC) (**Kahn et al., 2002*) Participants played an interactive competitive Domino game for fourteen minutes, in which they were instructed to take risks in order to win. This task was previously validated and performed in both healthy and clinical populations (Admon, Lubin, Rosenblatt, Stern, Kahn, Assaf, Hendler, 2013, Assaf, Kahn, Pearlson, Johnson, Yeshurun, Calhoun, Hendler, 2009, Ben-Zion, Shany, Admon, Keynan, Avisdris, Balter, Shalev, Liberzon, Hendler, Gonen, Admon, Podlipsky, Hendler, 2012, Hyatt, Assaf, Muska, Rosen, Thomas, Johnson, Hylton, Andrews, Reynolds, Krystal, et al., 2012, Kahn, Yeshurun, Rotshtein, Fried, Ben-Bashat, Hendler, 2002, Thaler, Gonen, Mirelman, Helmich, Gurevich, Orr-Urtreger, Bloem, Giladi, Hendler, et al., 2019). It involves decision-making (goal-conflict behavior), execution (risky vs. safe choice), anticipation (emotional regulation), and response to an outcome (punishment, non-punishment, reward, non-reward) and contains both visual and auditory cues. In this work, we examined the functional connectivity during the tasks, without considering the different conditions (i.e. without employing any hypothesis on the analysis).

### 2.3 fMRI acquisitions

For all participants, whole-brain functional and anatomical images were acquired using a 3.0 Tesla Siemens MRI system (MAGNETOM Prisma, Germany with a 20-channel head coil at our lab in Tel-Aviv Sourasky Medical Center. Functional images were acquired in an interleaved order (anterior to posterior), using a T2*-weighted gradient-echo planar imaging pulse sequence (TR/TE=2000/28ms, flip angle= 90${}^{\circ }$, voxel size 2.2 × 2.2 × 2.2mm, FOV=220 × 220mm, slice thickness=3mm, 36 slices per volume). A T1-weighted three-dimensional anatomical image was also collected, using a magnetization prepared rapid gradient echo (MPRAGE) sequence (TR/TE=2400/2.29ms, flip angle = 8${}^{\circ }$, voxel size 0.7× 0.7 × 0.7 mm, FOV = 224 × 224 mm), enabling optimal localization of the functional effects.

### 2.4 fMRI pre-processing

Pre-processing was performed using FMRIPREP 1.4 (Esteban et al., 2018), and further statistical analysis was performed using SPM12 (Friston, 2003). This process includes: (i) Slice time correction (ii) Head motion correction by six-parameter rigid body spatial transformations, (iii) A 4th-degree interpolation for detection and correction head motions (iv) Co-registration of the functional to corresponding structural maps using the normalized mutual information (NMI) objective, (v) Parcellation using the probabilistic Harvard-Oxford cortical and subcortical structural atlases (including 48 cortical and 21 subcortical areas, in both hemispheres; total of N=117 regions per participant), and (vi) Extraction of time courses for each participant individually. For each subject, activation levels of 117 brain areas were derived across task conditions. This time course included 300,450,195 samples (TR=2sec) for rs-fMRI, SRDC task and emotional reactivity task, respectively.

### 2.5 Analytics

Our analysis is conducted along four different axes. The first axis is predicting PTSD diagnosis at three different time-points. Here, the same model was trained on rs-fMRI and two different fMRI tasks. The second axis studies symptom based clusters of PTSD. We trained our model on rs-fMRI to identify the distinct classes of PTSD. In clinical use, it is beneficial to predict remission from PTSD. To this end, the third axis is a survival analysis within our prospective design, which predicts the chronicity of PTSD from T1 to T3. In particular, whether patients with PTSD at T1 still meet the same diagnostic criteria at T3. Finally, for the fourth axis, we applied statistical tests to identify pairwise correlations in our PTSD predicting model (the first axis) that discriminate between PTSD patients and controls.

All computational experiments employed a cross-validation scheme, in which five random splits of the dataset to train and test were created (number of participants in each time-point in training and test is reported in Table 6). In each iteration of the re-sampling procedure, 80% of the data were used as train and 20% as test. To benchmark our predictive model, we compared the model with recent ML and DL approaches by training all the baselines from scratch on our dataset, showing a significant improvement on all the fMRI tasks, both in classification accuracy, AUC and average precision. To demonstrate the contribution of the various components of our method and the different hyper-parameters in the network, we have conducted an ablation analysis in which we considered variants of the model and alternatives to several components of the network.

#### 2.5.1 Network architecture

**Fig. 2:**
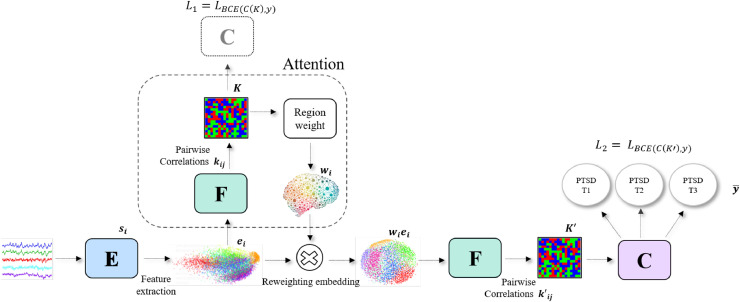
Illustration of the inference and train stages in PTSD diagnosis prediction: (i) At inference, given the time-series signal, the feature extractor E outputs an embedding for every brain region. The network F receives as input two concatenating embeddings and computes their connectivity score. Applying F to all the pairs of brain regions results in the connectivity matrix K. The attention mechanism computes region weight by aggregating the scores $k_{ij}$. The embedded brain regions features are updated by reweighting of the embedding $e_{i}$. A second connectivity matrix $K^{\prime }$ of pairwise correlations $k_{i}^{\prime }j$ is computed by F. Finally, prediction is performed by the classifier C. (ii) At training, the classification network C is used twice: once for $K^{\prime }$ and once for K.

The training dataset $X=\{\left(x_{1},y_{1}\right),\cdots \left(x_{n^{\prime }},y_{n^{\prime }}\right)\}$ consists of $n^{\prime }$ participants, where $x_{i}$ represents time-series signals and $y_{i}$ is a matching label in ${\left\{0,1\right\}}^{l}$ where l=3 for PTSD diagnosis prediction (see Section 2.5.4), l=20 for PTSD symptom clusters prediction (see Section 2.5.5) and l=1 for survival analysis (see Section 2.5.6). As mentioned in Section 2.4, each fMRI scan is parcellated into N=117 brain regions, each associated with a 1D sequence of T=300 data points for rs-fMRI, T=195 for emotional reactivity task, and T=450 for SRDC task. A single sample $x_{i}=\left(s_{1},s_{2},\cdots ,s_{N}\right)$ thus contains the information of multiple brain regions, where each brain region i is associated with a 1D sequence $s_{i}\in R^{T}$ obtained by aggregating the activations of this region. Our novel attention approach includes six stages presented in Fig. 2 and can be described by the following set of equations, which are applied to all regions i∈[N]:

1. Region embedding   Given time-series signal $s_{i}$, the feature extraction network E computes the region embedding $e_{i}\in R^{d}$ of the brain region $s_{i}$. Unless otherwise specified, we employ an embedding dimension of d=32:

(1) $$e_{i}=E(s_{i})$$

The feature extractor network E includes six 1D convolutional layers, each is followed by ReLU non-linearity. For all the convolutional layers, the kernel size is 3 and the padding is 1. The first convolutional layer includes 64 filters, the second 128 filters, the third and the fourth 256 filters, and the two last convolution layers include 512 filters. In addition to ReLU non-linearity, all the convolutional layers, except of the third and the fifth, are followed by Max pooling with kernel size 2 and stride of 2. The last layer is linear layer that outputs d features for every brain region.

2. Pairwise connectivity map   The similarity network F computes pairwise correlation $k_{ij}\in \left[0,1\right]$, represents the connection strength between brain regions embeddings $e_{i},e_{j}$:

(2) $$k_{i,j}=F(\left[e_{i},e_{j}\right])$$

where [a,b] is the concatenation of vectors a and b. We apply this network to all the different pairs of brain region embeddings, resulting in the connectivity matrix $K=\left[k_{ij}\right]\in R^{N\times N}$. The similarity network F is a MLP with three linear layers followed by a sigmoid.

3. Attention mechanism   The attention weight $w_{i}$ is computed by the relative importance of each outcoming interaction, as reflected by the pairwise correlations. Thus, the region attention weight is obtained for each region i, by aggregating the scores $k_{ij}$ across all other regions j:

(3) $$w_{i}=\beta +\left(1-\beta \right)\sum_{j\neq i}k_{ij}$$

where $k_{ij}$ is viewed as an attention value and β is a weighting parameter.

4. Reweighting embedding    The weights are used to produce a new set of brain region embeddings. The updated embedding $e_{i}^{\prime }=w_{i}e_{i}$ leverages the interactions of the region with other regions, i.e., reweighted embedding $w_{i}e_{i}$ is employed instead of the embedding $e_{i}$.

5. Updated connectivity map    Computing a second matrix of similarity scores $K^{\prime }=\left[k_{ij}^{\prime }\right]\in R^{N\times N}$. between the updated embeddings $e_{i}^{\prime }=w_{i}e_{i}$ and $e_{j}^{\prime }=w_{j}e_{j}$ using the same network F:

(4) $$k_{ij}^{\prime }=F(\left[w_{i}e_{i},w_{j}e_{j}\right])$$

where $k_{ij}^{\prime }\in \left[0,1\right]$. Similar forms of aggregations can be found in many attention schemes (Schwartz et al., 2019) (see Section 3 for further analysis).

6. Classification    Performing prediction by the classifier network C, based on the connectivity matrix $K^{\prime }$. The probability threshold for classification is set to 0.5.

(5) $$\bar{y}=C\left(k^{\prime }\right)\;where\;\bar{y}\in {\left\{0,1\right\}}^{l}$$

The classifier C has three linear layers, the first two are followed by ReLU and Dropout and the last one outputs l logits.

#### 2.5.2 Training objective

Training employs the binary cross entropy with logits multi-label loss (BCE), which treats each of the l labels as a separate binary classification problem. As presented in Fig. 2, It is used twice: once on the connectivity matrix K, using classifier C (Eq. (5)), and once on the updated matrix $K^{\prime }$. As mentioned in 2.5.1, $y,C\left(k\right)\in {\left\{0,1\right\}}^{l}$ where l=3 in PTSD diagnosis prediction, l=20 in PTSD symptom clusters prediction and l=1 in survival analysis.

(6) $$L_{1}=-\frac{1}{\left|X\right|}\sum_{(x,y)\in X}\left[y\log\sigma \left(C\left(k\right)\right)+\left(1-y\right)\log\left(1-\sigma \left(y,C\left(k\right)\right)\right)\right],$$

(7) $$L_{2}=-\frac{1}{\left|X\right|}\sum_{(x,y)\in X}\left[y\log\sigma \left(C\left(k^{\prime }\right)\right)+\left(1-y\right)\log\left(1-\sigma \left(y,C\left(k^{\prime }\right)\right)\right)\right]$$

(8) $$L=\lambda \cdot L_{1}+\left(1-\lambda \right)\cdot L_{2}\;,$$

where K and $K^{\prime }$ are functions of x (Eq. (2),(4)), σ is sigmoid function, and λ is a tradeoff parameter. To overcome in training the imbalance in label distribution described in Section 2.1, we used weight balancing which alters the weight that each training sample carries when computing the loss. For each time-point, the weight was proportional to the ratio between the number of subjects with PTSD and the total number of subjects in this time-point.

#### 2.5.3 Training hyperparameters

We used the Adam optimizer with β1=0.5, β2=0.999, and an initial learning rate of 0.0001. The learning rate is decreased by 10 every 30 epochs. In all experiments, the number of epochs is 100, we used a batch size of 20, loss weight λ=0.6 and attention value β=0.9.

#### 2.5.4 Predicting PTSD diagnosis

Our primary analysis concerned the prediction of PTSD diagnosis at three different time-points. This problem is treated as a three-label classification and the network architecture described in Section 2.5.1 is used with l=3. The training dataset $X=\{\left(x_{1},y_{1}\right),\cdots \left(x_{n^{\prime }},y_{n^{\prime }}\right)\}$ consists of $n^{\prime }$ participants, where $x_{i}$ represents time-series signals and $y_{i}$ is a matching label in ${\left\{0,1\right\}}^{3}$. Here, we treat each of the three time-points as a separate binary classification problem. Although we consider rs-fMRI to be the most suitable modality, to evaluate our model due to its ubiquitous nature and since it is not stimuli-dependent, we additionally trained the proposed architecture on different fMRI tasks, as mentioned in Section 2.2.

#### 2.5.5 Identifying unique PTSD symptom clusters

According to DSM-5, PTSD includes four symptom clusters: Intrusion (criteria B) consisting of five symptoms; Avoidance (criteria C)- two symptoms; Negative alternations in mood and cognition (criteria D)- seven symptoms; and Hyperaousal (criteria E)- six symptoms. The classification is based on PTSD diagnosis (by DSM-5): meeting criteria B if there is at least one of the five symptoms; criteria C- if at least one of the two symptoms, criteria D- if at least two of the seven symptoms; and criteria E- if at least two of the six symptoms.

To identify distinct, symptom-based clusters of PTSD, we trainrd our model, each time separately, on rs-fMRI dataset. The network architecture described in Section 2.5.1 is used independently for each time-point T1,T2,T3 with l=20. The training dataset $X=\{\left(x_{1},y_{1}\right),\cdots \left(x_{n^{\prime }},y_{n^{\prime }}\right)\}$ consists of $n^{\prime }$ participants, where $x_{i}$ represents time-series signals and $y_{i}$ is a matching label in ${\left\{0,1\right\}}^{20}$, for 20 symptoms in each time-point. In other words, for each time-point T1,T2 and T3, the label corresponding to each participant is a 20 dimensional binary vector ${\left\{0,1\right\}}^{20}$, such that each coordinate represents one PTSD symptom. The first five coordinates correspond to the five symptoms of criteria B, the next two to the symptoms of criteria C and so on. The probability threshold for each symptom is set to 0.5, and the classification to each cluster is based on DSM-5 clinical diagnosis. The results are reported in Section 3.2.

#### 2.5.6 Survival analysis

In clinical use, it is beneficial to predict remission from PTSD. Starting with a trauma-exposed group with high rates of PTSD at T1 (about 70%), we observe recovery over time (from T1 to T2, and from T2 to T3) resulting in a final group with mostly recovered healthy individuals and only a subset of PTSD patients (about 23%). We therefore aim to explore the chronicity of the PTSD symptoms from T1 to T3.

To this end, we trained our model to predict whether patients of each symptom cluster would still meet the same diagnostic criteria at T3. In this case, the network architecture described in Section 2.5.1 is used with l=1. The training dataset $X=\{\left(x_{1},y_{1}\right),\cdots \left(x_{n^{\prime }},y_{n^{\prime }}\right)\}$ consists of $n^{\prime }$ participants, where $x_{i}$ represents time-series signals and $y_{i}$ is a matching label in {0,1} indicating whether the patient still meet the same diagnosis at T3.

#### 2.5.7 Identifying discriminative connectivities

To identify discriminative connectivities, we compared the pairwise connectivities ($k_{ij}^{\prime }$) between participants with and without a diagnosis of PTSD, at each one of the three time-points. Independent t-tests were used to detect connectivities that significantly differed between the two groups, with a statistical threshold of alpha=0.05 and FDR-correction for multiple-comparisons (Benjamini and Hochberg, 1995). The results are reported in Section 3.4.

### 2.6 Ablation analysis

To demonstrate the advantage of learning a single representation for multiple time-points, we trained an alternative method that examines each time-point separately (a separate binary model is trained for each time-point). We performed an ablation analysis that demonstrates the contribution of the various components of our method, by employing multiple method variants of it. First, a model in which the network E is the identity transformation was tested, i.e. we replaced the 32 dimension embedding with the time-series signal input. The second variant we employed was a model in which F was replaced by a Pearson correlation. Last, to examine the contribution of attention mechanism, we considered several alternatives to our reweighting approach. (i) **A model based on the first connectivity map** This model includes only networks E,F and classifier C applied on matrix K. It is similar to deep FMRI (Riaz et al., 2018), however it differs in the architecture of the networks. (ii) **Graph convolutional neural network method as aggregation function** This model employs a graph convolutional neural network (Niepert et al., 2016) (GCN) instead of our proposed reweighting in Eq. (3). Based on a message passing neural network, the GCN method learns new representations by aggregating feature vectors of the neighboring regions. We tested several architectures and report the best one.

## 3 Results

### 3.1 PTSD diagnosis prediction

We report the mean and the standard deviation of the accuracy for each of the three time-points and prediction tasks as well as the AUC and average precision values from the prediction scores. The same experiments are also repeated when minimizing the mean squared error (MSE) for the CAPS score, instead of the binary PTSD classification. Since there is a high correlation between CAPS-IV and CAPS-5 total scores across all time-points (r>0.95), we report only CAPS-IV total scores in this section. CAPS-IV had been extensively used in neuroimaging studies of PTSD to date, and we report it here in order to keep continuity.

We consider two recently reported approaches: The deep fMRI (Riaz et al., 2018) network and a slightly modified version of the graph neural network approach of Li et al. (2019) (best effort reimplementation). Since Random Forest (RF) is a popular method in the literature for fMRI classification, we employed RF with 100 estimators (several options have been tested and the one with the best performance is reported), where the input was the concatenation of the data from all regions at all time-points.

The accuracy results of our model and baselines are reported in Table 2, Table 3, Table 4 for rs-fMRI, emotional reactivity task, and SRDC task (Kahn et al., 2002), respectively. Furthermore, the AUC binary classification rates, and average precision rates, for all the tasks and baselines are reported in Table 5, Table 6, respectively.

**Table 2:** Resting-state prediction. Accuracy of binary classification and MSE of CAPS (mean ±SD).

	Classification accuracy			CAPS-IV MSE		
Method	T1	T2	T3	T1	T2	T3
Deep FMRI Riaz et al. (2018)	74.0±6.6	59.0±12.0	43.4±9.0	0.5±0.2	0.5±0.2	0.3±0.2
Li et al. Li et al. (2019)	72.0±7.0	62.9±5.0	76.2±6.0	0.7±0.3	0.9±0.7	1.1±0.8
Random Forest (Li et al., 2019)	68.3±7.6	63.4±9.0	73.2±6.2	0.4±0.3	0.3 ±0.2	0.3 ±0.2
Raw data MLP	46.5±20.0	55.2±14.0	33.6±25.0	0.7±0.5	0.4±0.2	0.3±0.2
**Ours**	**88.6**±**2.0**	**80.4**±**3.0**	**84.0**±**5.4**	**0.2**±**0.0**	**0.3**±**0.1**	**0.1**±**0.1**
Ours + GCN	82.1±5.0	54.6±6.7	72.4±8.6	0.4±0.1	0.3±0.1	0.2±0.3
Ours 3 independent	81.0±3.0	77.0±3.0	80.0±1.0	0.9±0.2	0.4±0.1	0.1±0.0
Ours no reweighting	72.0±7.0	56.6±11.0	57.5±13.0	0.4±0.1	0.4±0.2	0.6±0.4
Ours Pearson as F	73.3±7.0	55.3±9.0	51.0±10.0	1.1±0.1	0.4±0.2	0.6±0.2
Ours E=identity	70.5±7.0	51.0±9.0	59.1±10.0	1.1±0.6	1.0±0.5	0.9±0.6

**Table 3:** Prediction based on Emotional reactivity task.

	Classification accuracy			CAPS-IV MSE		
Method	T1	T2	T3	T1	T2	T3
Deep FMRI (Riaz et al., 2018)	79.3±3.5	37.0±5.4	32.2±11.2	0.9±0.2	1.2±0.6	0.7±0.2
Li et al. Li et al. (2019)	79.8±4.6	64.0±8.1	70.8±13.5	1.1±0.1	1.3±0.4	0.9±0.5
Random Forest (Li et al., 2019)	74.9±3.7	59.0±12.0	68.5±7.1	1.0±0.1	1.4±0.4	1.0±0.1
Raw data MLP	74.9±6.5	60.9±5.8	59.7±5.1	1.3±0.3	1.5±0.3	1.0±0.3
**Ours**	**85.8**±**3.6**	**80.0**±**11.2**	**69.3**±**9.1**	**0.8**±**0.2**	**1.0**±**0.0**	**0.7**±**0.1**
Ours + GCN	84.1±2.4	69.8±7.0	66.5±9.1	0.9±0.4	1.4±0.4	0.9±0.1
Ours no reweighting	80.0±2.4	53.4±7.3	40.4±2.4	0.9±0.1	1.3±0.6	1.0±0.1
Ours Pearson as F	76.6±8.0	41.4±11.8	61.8±11.3	1.4±0.2	1.6±0.4	0.9±0.2
Ours E=identity	76.6±2.4	61.5±4.5	67.4±8.7	0.9±0.2	1.4±0.3	0.9±0.0

**Table 4:** Prediction based on Safe or Risky Domino Choice (SRDC) task.

	Classification accuracy			CAPS-IV MSE		
Method	T1	T2	T3	T1	T2	T3
Deep FMRI (Riaz et al., 2018)	74.1±2.4	30.0±9.6	22.7±4.6	0.9±0.0	0.8±0.2	0.6±0.1
Li et al. Li et al. (2019)	70.6±5.4	64.5±9.8	67.1±10.0	0.8±0.3	0.8±0.2	0.6±0.2
Random Forest (Li et al., 2019)	74.1±2.4	31.0±10.9	22.8±4.5	1.0±0.1	1.0±0.2	0.8±0.1
Raw data MLP	71.5±3.7	52.2±3.9	62.4±9.1	1.1±0.1	1.2±0.1	0.8±0.2
**Ours**	**80.7**±**1.8**	**68.3**±**10.0**	**70.4**±**7.5**	**0.8**±**0.1**	**0.7**±**0.2**	**0.6**±**0.1**
Ours + GCN	79.1±6.8	46.4±11.8	67.9±13.5	0.9±0.0	0.9±0.2	0.8±0.1
Ours no reweighting	79.2±1.6	55.3±10.5	58.6±11.8	0.9±0.1	1.2±0.2	0.8±0.2
Ours Pearson as F	67.5±4.6	47.1±16.8	52.1±13.6	1.5±0.6	2.0±0.6	1.1±0.3
Ours E=identity	66.7±8.2	44.2±11.0	52.6±5.6	1.0±0.2	1.1±0.2	0.8±0.1

**Table 5:** AUC of binary classification (mean ±SD).

	Resting state			Hariri			Domino		
Method	T1	T2	T3	T1	T2	T3	T1	T2	T3
Deep FMRI (Riaz et al., 2018)	0.51 ± 0.01	0.73 ± 0.08	0.69 ± 0.06	0.59 ± 0.11	0.70 ± 0.03	0.72 ± 0.04	0.53 ± 0.0	0.63 ± 0.05	0.63 ± 0.05
Li et al. Li et al. (2019)	0.69 ± 0.04	0.72 ± 0.03	0.74± 0.02	0.71 ± 0.07	0.75 ± 0.06	0.75 ± 0.02	0.70 ± 0.06	0.74 ± 0.02	0.76 ± 0.01
Random Forest (Li et al., 2019)	0.62± 0.08	0.70± 0.03	0.74± 0.01	0.57± 0.07	0.70± 0.03	0.72± 0.02	0.53± 0.05	0.69± 0.03	0.72± 0.01
Raw data MLP	0.70± 0.04	0.72± 0.02	0.72± 0.05	0.69± 0.04	0.72± 0.03	0.71± 0.03	0.65± 0.08	0.69± 0.02	0.68± 0.06
**Ours**	**0.80**±**0.05**	**0.73**±**0.02**	**0.76**±**0.01**	**0.80**±**0.03**	**0.80**±**0.02**	**0.76**±**0.01**	**0.76**±**0.03**	**0.76**±**0.04**	**0.76**±**0.01**
Ours+GCN	0.78 ± 0.09	0.69± 0.05	0.74± 0.02	0.77± 0.03	0.76± 0.03	0.72± 0.04	0.64± 0.08	0.72± 0.03	0.72± 0.02

**Table 6:** Average precision of binary classification (mean ±SD).

	Resting state			Hariri			Domino		
Method	T1	T2	T3	T1	T2	T3	T1	T2	T3
Deep FMRI (Riaz et al., 2018)	0.50±0.00	0.64±0.01	0.65±0.01	0.50±0.05	0.65±0.09	0.65±0.01	0.50±0.00	0.64±0.07	0.63±0.02
Li et al. Li et al. (2019)	0.71±0.03	0.68±0.04	0.68±0.03	0.60 ±0.08	0.66±0.04	0.66±0.04	0.61±0.04	0.67 ±0.04	0.71±0.07
Random Forest (Li et al., 2019)	0.53±0.03	0.65±0.02	0.66±0.02	0.52±0.01	0.65±0.01	0.66±0.00	0.49 ± 0.01	0.65±0.02	0.65±0.01
Raw data MLP	0.58±0.04	0.65±0.04	0.65±0.03	0.55±0.03	0.66±0.05	0.68±0.05	0.55±0.02	0.66±0.05	0.66±0.03
**Ours**	**0.77**±**0.03**	**0.73**±**0.00**	**0.76**±**0.04**	**0.71**±**0.03**	**0.76**±**0.05**	**0.77**±**0.01**	**0.71**±**0.02**	**0.67**±**0.02**	**0.79**±**0.05**
Ours+GCN	0.73±0.09	0.65±0.02	0.70±0.05	0.70±0.06	0.63±0.03	0.67±0.06	0.66±0.03	0.66±0.02	0.68±0.03

*Ablation analysis* As described in Section 2.6, we employed multiple variants for our method (see results in Table 2). First, we trained an alternative method that examines each time-point separately (a separate binary model was trained for each time-point). Although the alternative model yielded accurate rates of 81.0% in T1, 77.0% in T2 and 80.0% in T3, it can be seen from Table 2 that utilizing the prospective design to predict the dynamic PTSD diagnosis leads to a higher accuracy. The second variant, in which network E is the identity transformation, yielded accuracy rates of 70.5% in T1, 51.0% in T2 and 59.1% in T3, demonstrating the importance of using the feature extraction network E. Another variant of our model, in which F is replaced by a Pearson correlation, produced accuracy rates of 73.3% in T1, 51.0% in T2 and 59.1% in T3, demonstrating the importance of using the similarity network F to compute the pairwise connectivity map.

Furthermore, we considered several alternatives to our reweighting approach (see Section 2.6 for full details): (i) **A model based on the first connectivity map** This model yielded results of 72.0% in T1, 56.6% in T2 and 57.5% in T3, demonstrating the importance of updating the connectivity map K using an attention mechanism. (ii) **Graph convolutional neural network method as aggregation function** We can observe from Table 2, Table 3, Table 4, Table 5 the high accuracy and the high AUC values respectively, of the GCN variant on rs-fMRI data and all the tasks. In particular, we can observe high performance in T1 (Accuracy= 82.1%, AUC=0.78 on rs-fMRI, Accuracy=84.1%, AUC=0.77 on emotional reactivity task and Accuracy=79.1%, AUC=0.64 on SRDC task), and reasonably good performance on T3 (Accuracy=72.4%, AUC=0.74 on rs-fMRI, Accuracy=66.5%, AUC=0.72 on emotional reactivity task and Accuracy=67.9%, AUC=0.72 on SRDC task).

**Fig. 3:**
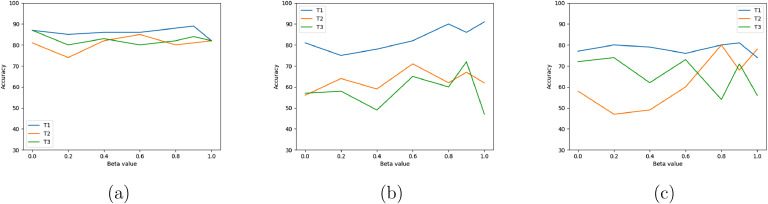
Sensitivity to parameter β of Eq. (3) (a) Resting-state task (b) Emotional reactivity task (c) SRDC task.

A parameter sensitivity analysis was performed by varying the value of the two parameters β (Eq. (3)) and λ (Eq. (8)) to validate the stability of the method. The results for the first parameter, given in Fig. 3, show that the method is largely insensitive to β for rs-fMRI, and more sensitive in the two other tasks. For λ=1, the loss L becomes $L_{1}$ (Eq. (6)), making use of the first connectivity map K only, even though the second connectivity map $K^{\prime }$ is still computed. However, at inference the classification is done by $K^{\prime }$.

**Fig. 4:**
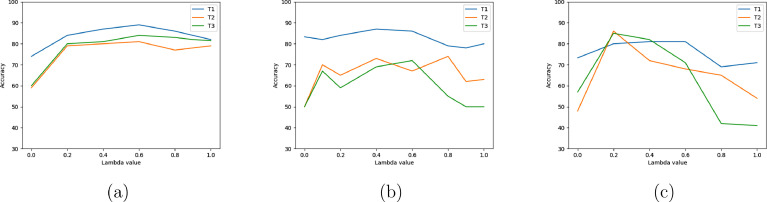
Sensitivity to the loss tradeoff λ in Eq. (8). (a) Resting-state task (b) Emotional reactivity task (c) SRDC task

Our method is largely stable to λ for the rs-fMRI data, with the exception of a slight trade-off between the accuracy at T2 and T3 (see Fig. 4). For the other tasks, increasing λ up to a certain point improves accuracy, but as λ increases beyond that point (≈ 0.2), the performance decreases. We note that for the emotional reactivity task and SRDC task, the optimal parameters are not the ones shown in the results tables, and that their performance can be improved (this is the result of selecting the parameters early on in the development process). Specifically, setting λ=0.2 in SRDC task highly improves the performance.

### 3.2 PTSD symptom cluster prediction

The classification accuracy rates as well as AUC values, for PTSD symptom cluster prediction, of our model and alternative Deep fMRI method (Riaz et al., 2018), are reported in Table 6. The number of participants at each time-point, including their division to different symptom clusters is reported in Table 8. In addition, the AUC for each symptom prediction is reported in Table 6. We obtain a high predictive ability for PTSD symptom cluster prediction with an average AUC= 0.81, average Accuracy=81.2% for class B; average AUC= 0.8, average Accuracy=73.0% for class C; average AUC= 0.78, average accuracy of 75.8% for class D. Finally, we obtain average AUC= 0.78 and average Accuracy=71.0% for class E (averaging on the three time-points).

**Fig. 5:**
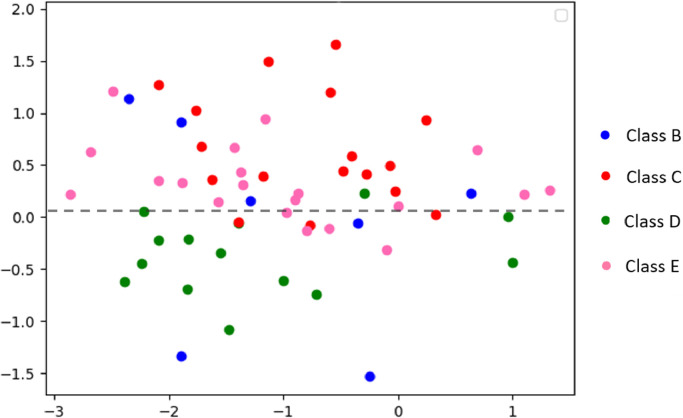
a t-SNE embedding of the last feature map before the classification to PTSD cluster. The color of each point represents the dominant cluster of the participant. There are 10,13,22,20 subjects with dominant symptoms of class B,C,D,E respectively.

In order to visualize the embedding space, we computed for each participant a dominant cluster of symptoms. This was determined by computing a cluster score for each cluster and participant (i.e. percentage of symptoms the participant meets out of all the symptoms in the cluster), normalizing the scores by considering the participant’s percentile among all participants and then choosing, if relevant, the most significant cluster for the participant. In other words, if there is no dominant cluster for the participant, we do not visualize the point and skip to the next participant. For full pseudo-code see Algo. 1. A visualization of the learned features, before the classification to a specific cluster is shown in Fig. 5. In particular, the features of each participant obtained from the first linear layer in the classifier C, that takes the connectivity matrix $K^{\prime }$ as input, flatten it into a single vector and projects it to feature vector of size 100. Each 100 dimensional feature point is embedded to two-dimensional point using t-SNE method (Maaten and Hinton, 2008), and the color of the visualized point is determined using the participant’s dominant cluster of symptoms.

### 3.3 Survival analysis

We used the survival analysis in our prospective design to predict persistence of PTSD. Our model was trained for predicting whether participants still meet the same diagnostic criteria at T3. The fraction of non-remitted participants at T3 was 28/115, i.e., out of 115 participants that met PTSD criteria at T1, 28 met the same criteria at T3.

The input to the subsequently trained predictive model was rs-fMRI data of participants, obtained one-month after trauma (T1), and the same cross-validation splits were employed as described in Section 3. A high predictive ability for chronicity of PTSD was obtained, with an AUC=0.84±0.02 and Accuracy= 81.33%±5.37.

### 3.4 Identifying discriminative connectivities

All significant findings of the *t*-test identifying the discriminative power of the pairwise similarities ($k_{ij}^{\prime }$) between PTSD and non-PTSD participants are given in the supplementary material, including both our results and those of the alternative models. Pairwise association analysis revealed several significant functional connectivity patterns, in line with previous PTSD neuroimaging literature, which are discussed below for each time-point.

#### 3.4.1 Neural measures at one-month post-trauma (T1) Clinical status at one-month post-trauma (T1)

**Fig. 6:**
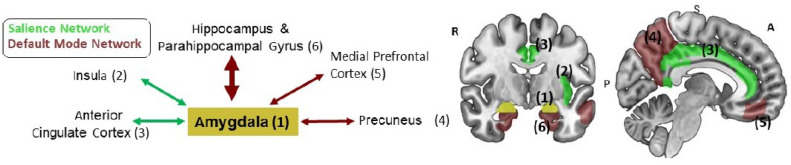
**Selected rs-fMRI connectivity patterns which significantly discriminated between participants with and without PTSD**. This figure presents the connectivity patterns of the amygdala which were significantly associated with PTSD diagnosis at T1. In particular, amygdala-parahippocampal connectivity at T1 also significantly discriminated between individuals with and without PTSD at T3 (marked with a bold arrow). Overall, these regions are part of two established large-scale brain networks: Salience Network (regions 1,2,3 in green) and Default-Mode Network (regions 4,5 and 6 in brown). Numbers 1–5 in the scheme on the left match to numbers 1–5 presented on the brain on the right.

*resting-state fMRI*  Fifty four significant rs-fMRI connectivities were found. Out of these, all but one includes the amygdala, a key brain region critically involved in emotional processing, both in the healthy population and in psychopathology (Davis, Whalen, 2001, Sripada, King, Garfinkel, Wang, Sripada, Welsh, Liberzon, 2012). Subset of amygdala connectivities are consistent with PTSD neuroimaging literature (see Fig. 6), including amygdalas functional connectivity with (i) bilateral-hippocampus, (ii) bilateral medial frontal cortices, (iii) right parahippocampal gyrus,(iv) left insular cortex, (v) bilateral precuneus, (vi) bilateral cingulate gyrus. Moreover, amygdala-parahippocampal gyrus connectivity at T1 also predicted PTSD diagnosis a year later (T3). *Emotional reactivity task*  Only three out of the 49 significant connectivities that were found involved the amygdala with (i) right superior temporal gyrus, (ii) left middle temporal gyrus, and (iii) left frontal pole. Nevertheless, as this is a visual processing task involving faces and shapes, we observed various connectivities at T1 known to be involved in visual processing (Hariri, Bookheimer, Mazziotta, 2000, Hendler, Rotshtein, Yeshurun, Weizmann, Kahn, Ben-Bashat, Malach, Bleich, 2003). For example, connectivities of the inferior temporal gyrus and connectivities of the occipital and temporo-occipital gyri. *SRDC task*  Two out of 99 significant connectivities that were found involved the amygdala, (i) right amygdala with right lateral occipital cortex (ii) left amygdala with right lateral occipital cortex. The rest of the connectivities are related to the nature of this dynamic task, as was shown in previous work (Admon, Lubin, Rosenblatt, Stern, Kahn, Assaf, Hendler, 2013, Ben-Zion, Shany, Admon, Keynan, Avisdris, Balter, Shalev, Liberzon, Hendler, Thaler, Gonen, Mirelman, Helmich, Gurevich, Orr-Urtreger, Bloem, Giladi, Hendler, et al., 2019). For example, visual processing involving occipital and inferior temporal areas, auditory processing involving heschls gyrus (Dierks et al., 1999) and frontal areas involve in decision making (Rushworth et al., 2011), anticipation and responses to risk and reward.

**Algorithm 1:**
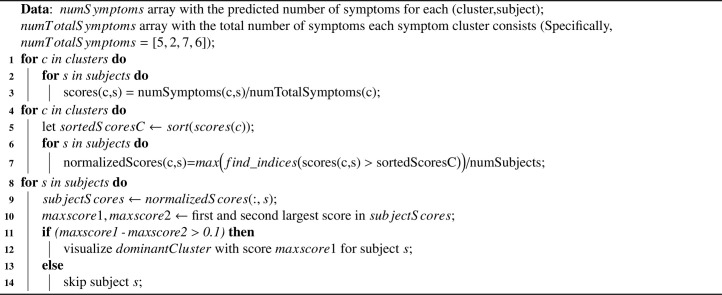
Symptom cluster Dominance calculation

#### 3.4.2 Neural measures at one-month post-trauma (T1) Clinical status at six-months post-trauma (T2)

*Resting-state fMRI (rs-fMRI)*  Four rs-fMRI significant connectivities were found. None of them is consistent with the neuroimaging literature of PTSD. *Emotion reactivity task*

As in T1, there are connectivities of the Inferior Temporal gyrus and temporo-occipital gyrus, which are related to the visual processing of this task (Miyashita, 1993). *SRDC Task*  The majority of the 135 significant connectivities that were found are highly relevant to the task (Admon, Lubin, Rosenblatt, Stern, Kahn, Assaf, Hendler, 2013, Ben-Zion, Shany, Admon, Keynan, Avisdris, Balter, Shalev, Liberzon, Hendler). Among these, 23 significant connectivities of the accumbens (known to be involved in the processing and analyzing rewarding stimuli) with different relevant regions, such as the hippocampus and parahippocampal (part of the Default Mode-Network) and 33 significant connectivities of the posterior cingulate cortex (related to intrinsic control networks and also part of the Default Mode-Network).

#### 3.4.3 Neural measures at one-month post-trauma (T1) Clinical status at fourteen-months post-trauma (T3)

*Resting-state fMRI (rs-fMRI)*  Seven significant connectivities were found, including one which is specifically relevant to PTSD: right amygdala with right parahippocampal gyrus. *Emotion reactivity task*  Out of 84 significant connectivities were found, only one involves the amygdala, namely amygdala-brain stem connectivity. Similar to T1 and T2, we observe the dominance of neural connectivities related to visual processing: Almost 50% of the connectivities involve the occipital (visual) cortex and 17 connectivities involve the fusiform cortex/gyrus, known to be the ǣface areaǥ (Grill-Spector, Knouf, Kanwisher, 2004, Kanwisher, McDermott, Chun, 1997). *SRDC task*  Out of the 115 significant connectivities that were found, seven connectivities involve the amygdala. Interestingly, ten connectivities involve the insula and nine connectivities invlove the anterior cingulate cortex, both are part of the Salience Network (Bonnelle et al., 2012). Moreover, hippocampus and parahippocampal gyrus are another dominant hubs for connectivities at this time-point (Raichle, 2015).

## 4 Discussion

Our novel classification method, across all fMRI scans and three time-points, demonstrated substantial improvement in accuracy, compared to all other methods recently suggested for fMRI analysis. We compared the model with previous ML and DL approaches by training all the baselines on the same dataset, showing a significant improvement on all the tasks, both in classification accuracy, AUC and average precision.

The first and primary axis of our work concerned the prediction of PTSD diagnosis at three different time-points. We considered rs-fMRI to be the most suitable modality as it does not have different conditions (in contrast to fMRI tasks). Nevertheless, we also trained the proposed architecture on different fMRI tasks, which provided further valid information on brain processing related to emotional reactivity, risky behavior, and sensitivity to punishments and rewards, as well as additional analyses pointing to the performance of our method in comparison to other approaches. Indeed, training the model on two different fMRI tasks, in addition to rs-fMRI data, may provide a more comprehensive understanding of neural activity in individuals with PTSD.

Here, we utilized the within-subject prospective design to predict a dynamic PTSD diagnosis for each patient, using neural measures obtained one-month after trauma and clinical status obtained one-, six- and fourteen months following the trauma. As demonstrated in Tab. 2, utilizing such a prospective design to predict the dynamic PTSD diagnosis leads to higher accuracy rates than the alternative model which examines each time-point separately. To demonstrate the contribution of the various components of our method and the different hyper-parameters in the network, we conducted an ablation analysis in which we considered several variants of the model and alternatives to the attention mechanism as well as to the functional connectivity network (see Section 3.1). Our model showed high performance in predicting PTSD diagnosis at three time-points, using fMRI scans only from the first time-point, when training both on rs-fMRI dataset (see Table 2) and on emotional reactivity task (see Table 3). Although the performance was lower on the SRDC task (see Table 4), it can be noted that our model still outperformed all other approaches.

Beyond the clinical dichotomous PTSD diagnosis (yes or no) and general symptom severity (as reflected by CAPS total scores), we assessed different PTSD symptom clusters in order to account for the known heterogeneity in symptom manifestation. This characterization could guide a more personalized clinical approach, for example by targeting the most dominant symptom cluster for each individual. The majority of studies found only qualitatively distinct profiles among diagnosed participants, and were conducted using the DSM-IV-TR symptom criteria or combined PTSD symptoms with additional items (e.g., adverse childhood experiences) (McLafferty et al., 2019). To date, Campbell et al. (2020) were the only study to examine latent classes with the DSM-5 criteria for PTSD, using linear and logistic regressions to identify demographic, trauma-related, and psychiatric characteristics associated with membership in each class.

In the second axis of our work, we filled this gap by training our model to identify distinct, symptom-based clusters of PTSD according to the most updated DSM-5 criteria. We obtained high predictive ability with an average AUC of around 0.8 for the different PTSD symptom clusters (across the three time-points) (see Table 7, Table 8). Furthermore, the classification accuracy and AUC in T1 was lower than in T2 and T3. This might be explained by the fact that 72% of the participants met PTSD diagnosis in T1, which according to DSM-5 means that they met the criteria for all PTSD clusters. However, in T2 and T3 there were more participants with unique symptom-based clusters without full PTSD diagnosis. As demonstrated by the visualization of the learned features before classifying to specific symptom cluster (see Fig. 5), participants in the center of the figure typically met the criteria for several different symptom clusters (although we visualized them only in one color representing the most dominant symptom criteria). Interestingly, there was a clear separation between participants with dominant symptoms of negative alternations in mood and cognition (green points, cluster D), and those with dominant avoidance symptoms (red points, cluster C). This suggests that symptom clusters C and D (avoidance, and negative alternations in mood and cognition, respectively) are highly separated, (i.e. both obtain unique features compared to the other clusters). This provides initial evidence, with possibly important clinical insights, regarding the ”real-world” validity of DSM-5 symptom clusters. In other words, the results support the transition from three clusters in DSM-IV to four clusters in DSM-5, and specifically the division into two distinct symptom clusters - avoidance, and negative alternations in mood and cognition (Kilpatrick, Resnick, Milanak, Miller, Keyes, Friedman, 2013, Pai, Suris, North, 2017).

**Table 7:** PTSD cluster predictions. Accuracy of Binary classification and AUC (mean ± SD).

			Classification accuracy				AUC			
			class B	class C	class D	class E	class B	class C	class D	class E
		T1	75.5 ± 7.8	69.5 ± 11.4	72.7 ± 11.0	69.8 ± 6.7	0.79 ± 0.08	0.84 ± 0.06	0.79 ± 0.06	0.82 ± 0.07
Ours		T2	86.6 ± 7.3	76.4 ± 4.2	78.5 ± 2.1	72.4 ± 1.4	0.79 ± 0.00	0.77 ± 0.05	0.75 ± 0.02	0.76 ± 0.01
		T3	81.4 ± 1.4	73.2 ± 3.5	76.1 ± 2.6	70.8 ± 3.6	0.85 ± 0.02	0.79 ± 0.03	0.79 ± 0.01	0.76 ± 0.01
		T1	59.1 ± 7.1	54.2 ± 1.3	59.1 ± 1.6	59.0 ± 2.5	0.54 ± 0.03	0.61 ± 0.06	0.55 ± 0.09	0.60 ± 0.06
Deep	fMRI	T2	71.0 ± 1.0	68.0 ± 2.1	66.0 ± 6.0	60.2 ± 0.2	0.75 ± 0.03	0.74 ± 0.04	0.72 ± 0.02	0.74 ± 0.02
		T3	64.4 ± 8.1	57.8 ± 8.8	59.0 ± 11.1	62.8 ± 6.3	0.67 ± 0.01	0.75 ± 0.01	0.68 ± 0.01	0.73 ± 0.02

**Table 8:** Mean AUC ± std for each PTSD symptom prediction.

The third axis of our work was the survival analysis. In clinical use, it is beneficial to predict remission or persistence of initial PTSD symptoms. Survival analysis studies the time to a dichotomous event (in this case, persistence of early PTSD symptoms), enabling group comparisons, accounting for censoring and inclusion of time-dependent covariates. Previous survival methods were conducted in PTSD populations mostly using single sampling and only demographic and clinical variables. For example, Müller et al. (2018) implemented a survival analytic approach from a lifetime perspective, to determine whether the expected duration of time until PTSD remission was related to a specific type of trauma, and to identify mediators of PTSD remission or persistence. In this work, we used the survival analysis in a prospective design to predict persistence of PTSD (from one-month to fourteen-months post-trauma), and obtained a high predictive ability for chronicity with an AUC of 0.84 and Accuracy of 81.3% for the general PTSD diagnosis.

Finally, for the fourth axis of our analysis, we applied statistical tests to identify pairwise correlations in the PTSD prediction model. The reported resting state discriminative connectivities are consistent with commonly found abnormal resting state functional connectivity patterns in PTSD involving amygdala functional connections (see Fig. 6) (Sripada et al., 2012a). Interestingly, several common resting-state functional connectivity patterns were found, including the amygdalas connectivity with core regions from the Salience Network and the Default Mode Network, which are known to be involved in emotional reactivity and emotional regulation, respectively. (Etkin et al., 2015). In contrast to rs-fMRI, less discriminative connectivities with the amygdala were found in the emotion reactivity task and SRDC task. This could be explained by the fact that while the rs-fMRI is one long scan, the two fMRI tasks involve dynamically changing conditions over time. In this work, in light of our data-driven approach, we examined the functional connectivity during the tasks without considering the different conditions. Still, task-based fMRI reveals process-related abnormalities such as fusiform connectivity with hippocampus/parahippocampus in the emotional reactivity task (related to emotional face processing) and nucleus accumbens connectivity with insula and parahippocampus in the SRDC task, possibly entails risk and reward processing (related to goal-directed behavior). This type of finding might gain robustness by adding hypothesis-driven analysis to the current data-driven approach. For example, examination of brain regions in specific functional contrasts (e.g., amygdala’s activity in the contrast of emotional faces vs. shapes) (Ben-Zion et al., 2020). It is worth noting that our resting state connectivity patterns were less significant at T2. Nevertheless, as was shown in previous work (Ben-Zion, Artzi, Niry, Keynan, Zeevi, et al., 2019, Ben-Zion, Shany, Admon, Keynan, Avisdris, Balter, Shalev, Liberzon, Hendler), it might be explained by the dynamic clinical manifestations during the first critical year after trauma (Hepp et al., 2008), in which an intermediary point of six-months might be too ”noisy” to isolate chronic PTSD symptom cluster. This is also supported by similar results in previous work examining structural abnormalities in this cohort (Ben-Zion et al., 2019a), as well as the lowered prediction results at Table 2, Table 3, Table 4 for T2.

Although our findings are promising and the cohort is relatively large for an fMRI study of clinical population, this work has several limitations that arise from the challenges of recruiting participants on a larger scale. First, there is class imbalance as 115/160, 39/135, 29/130 participants met PTSD diagnostic criteria at T1, T2 and T3, respectively. That is, most individuals recovered from initial symptoms after six- or fourteen-months from the traumatic incident. To overcome this challenge, we used weight-balancing during the training of the model. For each time-point, the weight was proportional to the ratio between the number of subjects with PTSD and the total number of subjects in this time-point. We also reported balanced error measures, such as the AUC. Second, the number of participants with PTSD decreased between the time-points and at T3 there were only 29 participants with PTSD. Splitting the data to 80% training and 20% test resulted in 23 participants with PTSD in the training set, and 6 in the test set. While this might have reduced the statistical power of the results, we repeated the experiment five times (in a cross validation scheme) in order to somewhat mitigate this issue. Lastly, in this work we used fMRI scans at T1 in order to predict PTSD symptoms at T2 and T3, thus precluding mechanistic insights regarding the clinical outcome. Future work should consider longitudinal neuroimaging and neuropsychological measures from all three time-points to enhance explainability of the clinical prediction. This could possibly guide therapeutic approaches that are more mechanistic in nature.

## 5 Conclusions

Our work demonstrates a computational approach for identifying objective variables linked to clinical PTSD diagnosis across different time-points during the first critical year following trauma exposure, based on single-session examination of functional connectivity shortly after exposure to trauma. To this end, we applied a novel deep learning model that employs fMRI scans, obtained shortly after trauma, to predict PTSD symptoms at one-, six- and fourteen-months following trauma. Our method demonstrates a significant improvement in performance in comparison to other analytical techniques reported in fMRI literature. The prediction is highly accurate compared to the existing methods for all three time-points and benefits from learning these all at once, using a single model. We further show a high predictive ability for predicting PTSD symptom clusters and persistence of the disorder. If validated, the objective features derived from our computational model may further guide mechanism-driven interventions for PTSD (e.g., neuromodulation techniques). Our code will be promptly shared as an open-source code.

## Ethical approval

The research study meets all ethical regulations as required by NYU Langone Health Institutional Review Board (IRB) and ethics committee in Tel-Aviv Sourasky Medical Center (Reference number 0207/14). All subjects gave written informed consent in accordance with the Declaration of Helsinki. This study is registered at ClinicalTrials.gov (registration number: NCT03756545)

## CRediT authorship contribution statement

**Shelly Sheynin:** Conceptualization, Methodology, Software, Formal analysis, Investigation, Writing - original draft, Writing - review & editing. **Lior Wolf:** Conceptualization, Methodology, Writing - original draft, Writing - review & editing, Supervision. **Ziv Ben-Zion:** Methodology, Resources, Data curation, Writing - original draft, Writing - review & editing. **Jony Sheynin:** Methodology, Resources, Data curation, Writing - review & editing. **Shira Reznik:** Resources, Software. **Jackob Nimrod Keynan:** Resources, Data curation. **Roee Admon:** Resources. **Arieh Shalev:** Conceptualization, Methodology, Funding acquisition, Writing - original draft, Writing - review & editing. **Talma Hendler:** Conceptualization, Methodology, Resources, Funding acquisition, Writing - original draft, Writing - review & editing. **Israel Liberzon:** Conceptualization, Methodology, Resources, Funding acquisition, Writing - original draft, Writing - review & editing.

## Appendix A Supplementary materials

Supplementary Data S1 Supplementary Raw Research Data. This is open data under the CC BY license http://creativecommons.org/licenses/by/4.0/ Supplementary Data S1
