# Supplementary material for: Deep learning model of fMRI connectivity predicts PTSD symptom trajectories in recent trauma survivors
Source: Neuroimage. 2021 Sep;238:118242. doi: 10.1016/j.neuroimage.2021.118242 (PMC8350148; doi:10.1016/j.neuroimage.2021.118242)
Supplement: Supplementary Data S1 — Supplementary Raw Research Data. This is open data under the CC BY license http://creativecommons.org/licenses/by/4.0/ [file mmc1.pdf]

Resting-state significant connections for PTSD prediction at T1

*Format: region A, region B (p-value); sorted by significance.*

| Our Model | Ours E is the identity | Deep fMRI | Ours + GCN |
|---|---|---|---|
| Left Occipital Fusiform Gyrus, Left Amygdala ($3.1264\times10^{-6}$) | Left Insular Cortex, Left Central Opercular Cortex (0.0002) | Left Middle Temporal Gyrus anterior division, Left Frontal Medial Cortex ($6.3516\times10^{-6}$) | Right Middle Frontal Gyrus, Left Planum Polare ($7.7139\times10^{-7}$) |
| Left Frontal Operculum Cortex, Left Amygdala ($4.8752\times10^{-6}$) | | | Right Inferior Frontal Gyrus pars opercularis, Left Superior Temporal Gyrus, anterior division ($6.5849\times10^{-6}$) |
| Left Precuneous Cortex, Left Amygdala ($4.9943\times10^{-6}$) | | | |
| Right Temporal Pole, Left Amygdala ($8.0233\times10^{-6}$) | | | |
| Left Lingual Gyrus, Left Amygdala ($1.1271\times10^{-5}$) | | | |
| Left Superior Parietal Lobule, Left Amygdala ($1.3127\times10^{-5}$) | | | |
| Left Supramarginal Gyrus anterior division, Left Amygdala ($1.7773\times10^{-5}$) | | | |
| Right Supracalcarine Cortex, Left Amygdala ($1.8911\times10^{-5}$) | | | |
| Left Superior Temporal Gyrus anterior division, Left Amygdala ($2.9575\times10^{-5}$) | | | |
| Left Temporal Pole, Left Amygdala ($2.9890\times10^{-5}$) | | | |
| Left Middle Temporal Gyrus anterior division, Left Amygdala ($3.4741\times10^{-5}$) | | | |
| Right Lingual Gyrus, Left Amygdala ($3.7520\times10^{-5}$) | | | |
| Left Middle Temporal Gyrus temporooccipital part, Left Amygdala ($5.1038\times10^{-5}$) | | | |
| Right Precuneous Cortex, Left Amygdala ($5.3208\times10^{-5}$) | | | |
| Right Cingulate Gyrus posterior division, Left Amygdala ($5.4696\times10^{-5}$) | | | |
| Right Middle Temporal Gyrus anterior division, Left Amygdala ($6.0990\times10^{-5}$) | | | |
| Left Frontal Medial Cortex, Left Amygdala ($6.8013\times10^{-5}$) | | | |
| Left Middle Frontal Gyrus, Left Amygdala ($7.6173\times10^{-5}$) | | | |
| Left Heschl's Gyrus, Left Amygdala ($7.9843\times10^{-5}$) | | | |
| Right Frontal Operculum Cortex, Left Amygdala ($8.5054\times10^{-5}$) | | | |
| Left Precentral Gyrus, Left Amygdala ($8.5142\times10^{-5}$) | | | |
| Left Pallidum, Left Amygdala ($8.7152\times10^{-5}$) | | | |
| Left Postcentral Gyrus, Left Amygdala ($9.8543\times10^{-5}$) | | | |
| Left Cingulate Gyrus posterior division, Left Amygdala ($9.8831\times10^{-5}$) | | | |
| Left Thalamus, Left Amygdala (0.0001) | | | |
| Left Supramarginal Gyrus, posterior division, Left Amygdala (0.0001) | | | |
| Right Frontal Medial Cortex, Left Amygdala (0.0001) | | | |
| Left Inferior Frontal Gyrus pars triangularis, Left Amygdala (0.0001) | | | |
| Right Intracalcarine Cortex, Left Amygdala (0.0001) | | | |
| Left Intracalcarine Cortex, Left Amygdala (0.0002) | | | |
| Right Thalamus, Left Amygdala (0.0002) | | | |
| Left Hippocampus, Left Amygdala (0.0002) | | | |
| Right Central Opercular Cortex, Left Amygdala (0.0002) | | | |
| Right Frontal Orbital Cortex, Left Amygdala (0.0002) | | | |
| Right Temporal Fusiform Cortex posterior division, Left Amygdala (0.0002) | | | |
| Right Superior Parietal Lobule, Left Amygdala (0.0002) | | | |
| Right Hippocampus, Left Amygdala (0.0002) | | | |
| Right Putamen, Left Amygdala (0.0002) | | | |
| Left Insular Cortex, Left Amygdala (0.0002) | | | |
| Left Subcallosal Cortex, Left Amygdala (0.0002) | | | |
| Right Inferior Temporal Gyrus anterior division, Left Amygdala (0.0002) | | | |
| Left Cerebral Cortex, Left Amygdala (0.0002) | | | |
| Right Inferior Frontal Gyrus pars triangularis, Left Amygdala (0.0003) | | | |
| Right Parahippocampal Gyrus anterior division, Left Amygdala (0.0003) | | | |
| Left Putamen, Left Amygdala (0.0003) | | | |
| Left Supracalcarine Cortex, Left Amygdala (0.0003) | | | |
| Right Parahippocampal Gyrus posterior division, Left Amygdala (0.0003) | | | |
| Right Amygdala, Left Amygdala (0.0003) | | | |
| Right Pallidum, Left Amygdala (0.0003) | | | |
| Left Planum Temporale, Left Amygdala (0.0003) | | | |
| Left Middle Temporal Gyrus anterior division, Left Hippocampus (0.0003) | | | |
| Right Superior Temporal Gyrus anterior division, Left Amygdala (0.0003) | | | |
| Left Cerebral White Matter, Left Amygdala (0.0003) | | | |

Resting-state significant connections for PTSD prediction at T2

*Format: region A, region B (p-value); sorted by significance.*

| Our Model | Ours E is the identity | Deep fMRI | Ours + GCN |
|---|---|---|---|
| Left Inferior Frontal Gyrus pars triangularis, Left Superior Temporal Gyrus anterior division ($2.5285\times10^{-6}$) | None | None | Left Superior Temporal Gyrus anterior division, Right Occipital Pole ($1.2829\times10^{-7}$) |
| Right Inferior Temporal Gyrus posterior division, Right Temporal Fusiform Cortex posterior division ($2.4331\times10^{-6}$) | | | Right Inferior Temporal Gyrus posterior division, Right Lateral Occipital Cortex inferior division ($5.1671\times10^{-6}$) |
| Left Temporal Occipital Fusiform Cortex, Left Superior Temporal Gyrus anterior division ($7.8789\times10^{-6}$) | | | |
| Left Lateral Ventrical, Left Superior Temporal Gyrus anterior division ($1.9476\times10^{-5}$) | | | |

Resting-state significant connections for PTSD prediction at T3

*Format: region A, region B (p-value); sorted by significance.*

| Our Model | Ours E is the identity | Deep fMRI | Ours + GCN |
|---|---|---|---|
| Right Amygdala, Right Parahippocampal Gyrus posterior division ($2.0964\times10^{-7}$) | Right Middle Temporal Gyrus posterior division, Right Frontal Orbital Cortex ($5.0969\times10^{-7}$) | Right Temporal Fusiform Cortex posterior division, Left Putamen ($3.0758\times10^{-6}$) | Left Lateral Occipital Cortex superior division, Right Temporal Fusiform Cortex anterior division ($2.3978\times10^{-8}$) |
| Right Temporal Fusiform Cortex anterior division, Right Parahippocampal Gyrus posterior division, ($2.8977\times10^{-5}$) | Left Inferior Temporal Gyrus anterior division, Left Frontal Operculum Cortex ($7.9054\times10^{-7}$) | Left Middle Temporal Gyrus temporooccipital part, Left Paracingulate Gyrus ($7.0893\times10^{-6}$) | Left Precuneous Cortex, Right Thalamus ($6.3765\times10^{-6}$) |
| Left Superior Parietal Lobule, Left Intracalcarine Cortex ($3.8247\times10^{-5}$) | Left Inferior Temporal Gyrus anterior division, Right Occipital Pole ($1.0158\times10^{-6}$) | Left Parahippocampal Gyrus posterior division, Left Putamen ($1.3051\times10^{-5}$) | Left Lateral Occipital Cortex superior division, Left Temporal Fusiform Cortex anterior division ($1.4664\times10^{-6}$) |
| Right Superior Parietal Lobule, Left Intracalcarine Cortex ($4.1768\times10^{-5}$) | Right Temporal Fusiform Cortex anterior division, Right Occipital Pole ($1.4489\times10^{-6}$) | | Right Occipital Pole, Right Thalamus ($4.1133\times10^{-6}$) |
| Left Heschl's Gyrus, Right Parahippocampal Gyrus posterior division ($1.1620\times10^{-5}$) | Right Subcallosal Cortex, Right Occipital Pole ($2.1227\times10^{-6}$) | | Left Supramarginal Gyrus posterior division, Right Temporal Fusiform Cortex anterior division ($6.8209\times10^{-6}$) |
| Left Temporal Fusiform Cortex posterior division, Right Cingulate Gyrus anterior division ($1.4193\times10^{-5}$) | Left Inferior Temporal Gyrus temporooccipital part, Right Occipital Pole ($3.6727\times10^{-6}$) | | Left Supramarginal Gyrus posterior division, Right Frontal Medial Cortex ($1.8186\times10^{-5}$) |
| Right Planum Polare, Right Parahippocampal Gyrus posterior division ($1.9696\times10^{-5}$) | Right Middle Temporal Gyrus posterior division, Right Supramarginal Gyrus posterior division ($5.8505\times10^{-6}$) | | Left Occipital Fusiform Gyrus, Right Planum Temporale ($3.589\times10^{-5}$) |
| | Left Temporal Fusiform Cortex anterior division, Right Occipital Pole ($6.1066\times10^{-6}$) | | Left Intracalcarine Cortex, Left Temporal Fusiform Cortex anterior division ($4.3293\times10^{-5}$) |
| | Left Inferior Temporal Gyrus posterior division, Right Supramarginal Gyrus posterior division ($6.4291\times10^{-6}$) | | Left Precuneous Cortex, Left Caudate ($6.2120\times10^{-5}$) |
| | Right Frontal Medial Cortex, Right Frontal Operculum Cortex ($6.7027\times10^{-6}$) | | Left Parietal Operculum Cortex, Left Heschl's Gyrus ($7.4230\times10^{-5}$) |
| | | | Right Amygdala, Left Temporal Pole ($7.8668\times10^{-5}$) |
| | | | Left Supramarginal Gyrus posterior division, Brain-Stem ($8.5288\times10^{-5}$) |
| | | | Right Amygdala, Right Cerebral White Matter ($8.7962\times10^{-5}$) |

Emotional reactivity task significant connections for PTSD prediction at T1

*Format: region A, region B (p-value); sorted by significance.*

| Our Model | Ours E is the identity | Deep fMRI | Ours + GCN |
|---|---|---|---|
| Left Inferior Temporal Gyrus, posterior division', "Right Heschl's Gyrus (5.6014e-10) | Right Juxtapositional Lobule Cortex (formerly Supplementary Motor Cortex), Left Accumbens (1.5036e-06) | Right Temporal Occipital Fusiform Cortex, Left Insular Cortex (5.4468e-06) | Right Insular Cortex, Right Inferior Temporal Gyrus, anterior division (8.2923e-08) |
| Left Insular Cortex', 'Left Frontal Medial Cortex (2.4024e-09) | Right Juxtapositional Lobule Cortex (formerly Supplementary Motor Cortex), Right Paracingulate Gyrus (3.7196e-06) | Left Insular Cortex, Right Temporal Occipital Fusiform Cortex (5.4468e-06) | Right Inferior Frontal Gyrus, pars triangularis, Right Inferior Temporal Gyrus, anterior division (3.5266e-07) |
| Right Planum Polare', 'Left Superior Frontal Gyrus (2.5430e-09) | Right Juxtapositional Lobule Cortex (formerly Supplementary Motor Cortex), Right Pallidum (1.1173e-05) | Right Parahippocampal Gyrus, Right Temporal Occipital Fusiform Cortex (1.94702e-05) | Right Parahippocampal Gyrus, anterior division, Right Inferior Temporal Gyrus, anterior division (3.6805e-07) |
| Left Inferior Temporal Gyrus, posterior division', "Left Heschl's Gyrus (includes H1 and H2)") (5.938e-09) | Left Temporal Occipital Fusiform Cortex, Left Accumbens (1.244e-05) | Right Temporal Occipital Fusiform Cortex, Right Parahippocampal Gyrus, (1.94702e-05) | Right Frontal Operculum Cortex, Right Inferior Temporal Gyrus, anterior division (1.01336e-06) |
| Left Planum Temporale', 'Right Supramarginal Gyrus, anterior division (1.04920e-08) | Right Accumbens, Right Paracingulate Gyrus (1.43949e-05) | Right Lateral Occipital Cortex, Right Superior Parietal Lobule (3.0756e-05) | Right Cerebral Cortex , Right Inferior Temporal Gyrus, anterior division (3.9241e-06) |
| Right Planum Polare', 'Right Lateral Ventricle (1.05916e-08) | Right Frontal Orbital Cortex, Right Paracingulate Gyrus (1.70745e-05) | Right Superior Parietal Lobule, Right Lateral Occipital Cortex (3.0756e-05) | Right Inferior Frontal Gyrus, pars opercularis, Right Inferior Temporal Gyrus, anterior division (4.7076e-06) |
| Left Parietal Operculum Cortex', 'Right Angular Gyrus (1.40373e-08) | Left Temporal Occipital Fusiform Cortex, Left Planum Temporale (1.77245e-05) | Right Temporal Occipital Fusiform Cortex, Right Lingual Gyrus (4.1263e-05) | Right Frontal Pole, Right Inferior Temporal Gyrus, anterior division (5.35546e-06) |
| Left Parietal Operculum Cortex', 'Right Middle Temporal Gyrus, posterior division (1.539e-08) | Left Juxtapositional Lobule Cortex (formerly Supplementary Motor Cortex), Right Planum Polare (1.95765e-05) | Right Lingual Gyrus, Right Temporal Occipital Fusiform Cortex (4.1263e-05) | Right Postcentral Gyrus, Right Inferior Temporal Gyrus, anterior division (5.3761e-06) |
| Right Middle Temporal Gyrus, anterior division', 'Right Precuneous Cortex (1.65276e-08) | Right Hippocampus, Right Paracingulate Gyrus (2.11504e-05) | Left Cingulate Gyrus, anterior division, Right Temporal Occipital Fusiform Cortex (6.4363e-05) | Right Inferior Frontal Gyrus, pars triangularis, Right Temporal Fusiform Cortex, anterior division (5.4462e-06) |
| Left Insular Cortex', 'Right Precuneous Cortex (1.950e-08) | Left Thalamus, Left Accumbens (2.11863e-05) | Right Temporal Occipital Fusiform Cortex, Left | Left Frontal Orbital Cortex, Right Inferior Temporal Gyrus, anterior division (6.0387e-06) |

| | | | |
|---|---|---|---|
| | | Cingulate Gyrus, anterior division (6.4363e-05) | |
| Right Superior Temporal Gyrus, posterior division', 'Right Planum Polare (2.39404e-08) | Right Juxtapositional Lobule Cortex (formerly Supplementary Motor Cortex), Right Intracalcarine Cortex (2.30135e-05) | Right Temporal Occipital Fusiform Cortex, Left Supramarginal Gyrus, (7.7745e-05) | Right Amygdala, Right Inferior Temporal Gyrus, anterior division (7.3030e-06) |
| Left Planum Temporale', 'Right Middle Frontal Gyrus (2.64413e-08) | Right Juxtapositional Lobule Cortex (formerly Supplementary Motor Cortex), Left Supracalcarine Cortex (2.4403e-05) | Left Supramarginal Gyrus, anterior division, Right Temporal Occipital Fusiform Cortex (7.7745e-05) | Right Inferior Frontal Gyrus, pars triangularis, Left Inferior Temporal Gyrus, temporooccipital part (7.956e-06) |
| Right Juxtapositional Lobule Cortex (formerly Supplementary Motor Cortex)', 'Left Precuneous Cortex (2.7939e-08) | Left Temporal Occipital Fusiform Cortex, Left Temporal Fusiform Cortex, anterior division (2.466e-05) | Left Supramarginal Gyrus, anterior division, Right Lateral Occipital Cortex, inferior division (8.1190e-05) | Right Inferior Frontal Gyrus, pars triangularis, Right Temporal Fusiform Cortex, posterior division (8.339e-06) |
| Right Planum Polare', 'Left Frontal Pole (4.35055e-08) | Left Occipital Pole, Left Planum Temporale (2.587e-05) | Right Lateral Occipital Cortex, inferior division, Left Supramarginal Gyrus, (8.1190e-05) | Right Frontal Operculum Cortex, Right Temporal Fusiform Cortex, anterior division (9.5952e-06) |
| Left Inferior Temporal Gyrus, posterior division', 'Left Superior Temporal Gyrus, posterior division (4.4632e-08) | Left Inferior Temporal Gyrus, posterior division, Left Accumbens (2.58e-05) | Right Insular Cortex, Right Temporal Occipital Fusiform Cortex (9.7008e-05) | Right Juxtapositional Lobule Cortex (formerly Supplementary Motor Cortex), Right Inferior Temporal Gyrus, anterior division (1.13664e-05) |
| Left Parietal Operculum Cortex', 'Right Precuneous Cortex (4.8249e-08) | Left Juxtapositional Lobule Cortex (formerly Supplementary Motor Cortex), Right Superior Parietal Lobule (2.7911e-05) | Right Temporal Occipital Fusiform Cortex, Right Insular Cortex (9.7008e-05) | Right Inferior Frontal Gyrus, pars triangularis, Left Parahippocampal Gyrus, anterior division (1.5364e-05) |
| Right Subcallosal Cortex', 'Left Pallidum (4.9840e-08) | Right Supramarginal Gyrus, posterior division, Right Hippocampus (2.8319e-05) | Left Temporal Fusiform Cortex, posterior division, Right Temporal Occipital Fusiform Cortex (0.0001) | Right Frontal Operculum Cortex, Left Inferior Temporal Gyrus, temporooccipital part (2.11595e-05) |
| Left Planum Temporale', 'Right Cuneal Cortex (6.5965e-08) | Right Supramarginal Gyrus, posterior division, Right Inferior Temporal Gyrus, temporooccipital part (2.856e-05) | Right Temporal Occipital Fusiform Cortex, Left Temporal Fusiform Cortex, posterior division (0.0001) | Right Cingulate Gyrus, anterior division, Right Inferior Temporal Gyrus, anterior division (2.363e-05) |
| Left Insular Cortex', 'Right Frontal Medial Cortex (1.1328e-07) | Right Accumbens, Left Accumbens (2.8870e-05) | Right Inferior Temporal Gyrus, posterior division, Right Temporal Occipital Fusiform Cortex (0.0001) | Right Superior Parietal Lobule, Right Inferior Temporal Gyrus, anterior division (2.46626e-05) |
| Left Planum Temporale', 'Right Middle Temporal Gyrus, temporooccipital part (1.40948e-07) | Left Amygdala, Left Planum Temporale (2.9516e-05) | Right Temporal Occipital Fusiform Cortex, Right Inferior Temporal Gyrus, (0.0001) | Left Cerebral Cortex , Right Inferior Temporal Gyrus, anterior division (2.7360e-05) |
| Right Planum Polare', 'Left Frontal Medial Cortex (1.49110e-07) | Left Juxtapositional Lobule Cortex, Right Inferior Temporal Gyrus, anterior division (3.289e-05) | Left Superior Parietal Lobule, Right Lateral Occipital Cortex (0.0001) | Right Insular Cortex, Right Temporal Fusiform Cortex, anterior division (2.8618e-05) |
| Right Superior Temporal Gyrus, posterior division', 'Right Amygdala (1.83033e-07) | Left Temporal Occipital Fusiform Cortex, Right Inferior Temporal Gyrus, anterior division (4.069e-05) | Right Lateral Occipital Cortex, inferior division, Left Superior Parietal Lobule (0.0001) | Right Frontal Operculum Cortex, Right Temporal Fusiform Cortex, posterior division (3.2195e-05) |
| Right Inferior Temporal Gyrus, posterior division', 'Left Parietal Operculum Cortex (1.94058e-07) | Right Juxtapositional Lobule Cortex (formerly Supplementary Motor Cortex), Right Putamen (4.493e-05) | | Right Supramarginal Gyrus, posterior division, Right Inferior Temporal Gyrus, (3.4146e-05) |
| Right Superior Temporal Gyrus, posterior division', 'Right Inferior Temporal Gyrus, temporooccipital part (2.20872e-07) | Right Juxtapositional Lobule Cortex (formerly Supplementary Motor Cortex), Right Thalamus (4.857e-05) | | Left Lateral Occipital Cortex, inferior division, Right Inferior Temporal Gyrus, anterior division (3.9696e-05) |
| Right Cerebral Cortex ', 'Left Precuneous Cortex (2.27383e-07) | Right Juxtapositional Lobule Cortex (formerly Supplementary Motor Cortex), Right Planum Polare (4.96e-05) | | Left Middle Temporal Gyrus, posterior division, Right Inferior Temporal Gyrus, (4.20746e-05) |
| Right Subcallosal Cortex', 'Right Caudate (3.1441e-07) | Right Occipital Pole, Left Planum Temporale (5.188e-05) | | Right Frontal Operculum Cortex, Left Lateral Ventrical (4.2547e-05) |
| Left Temporal Fusiform Cortex, anterior division', 'Right Supramarginal Gyrus, [illegible] | Right Juxtapositional Lobule Cortex, Right Frontal Pole (6.5181e-05) | | Left Temporal Fusiform Cortex,, Right Inferior Temporal Gyrus, (4.510e-05) |
| Right Caudate', 'Left Lateral Occipital Cortex, superior division (3.6050e-07) | Right Juxtapositional Lobule Cortex), Right Lingual Gyrus (7.2323e-05) | | Right Parahippocampal Gyrus, posterior division, Right Inferior Temporal Gyrus, (4.6355e-05) |
| Left Inferior Temporal Gyrus, , 'Left Planum Temporale (3.72753e-07) | Right Juxtapositional Lobule Cortex, Right Inferior Temporal Gyrus, anterior division (7.2646e-05) | | Left Middle Temporal Gyrus, temporooccipital part, Right Inferior Temporal Gyrus, (4.928e-05) |
| Left Accumbens', 'Left Cingulate Gyrus, anterior division (4.0955e-07) | Right Occipital Pole, Left Caudate (7.3954e-05) | | Right Inferior Temporal Gyrus, Right Juxtapositional Lobule Cortex (5.2902e-05) |
| Right Superior Temporal Gyrus, 'Right Lateral Ventricle (6.8358e-07) | Right Temporal Occipital Fusiform Cortex, Left Accumbens (7.5082e-05) | | Right Insular Cortex, Right Cuneal Cortex (5.5402e-05) |
| Left Accumbens', 'Right Precentral Gyrus (7.9114e-07) | Left Juxtapositional Lobule Cortex (), Right Paracingulate Gyrus (7.7757e-05) | | Right Angular Gyrus, Right Inferior Temporal Gyrus, anterior division (5.5599e-05) |
| Left Insular Cortex', 'Right Middle Temporal Gyrus, posterior division (9.1287e-07) | Left Temporal Fusiform Cortex, posterior division, Left Accumbens (7.925e-05) | | Right Precentral Gyrus, Right Inferior Temporal Gyrus, anterior division (5.64681e-05) |
| Right Parahippocampal Gyrus, anterior division', 'Right Precentral Gyrus (9.4955e-[illegible] | Left Inferior Temporal Gyrus, posterior division, Right Caudate (7.9710e-05) | | Right Putamen, Right Inferior Temporal Gyrus, anterior division (6.0162e-05) |
| Left Middle Temporal Gyrus, 'Right Amygdala (1.05986e-06) | Right Thalamus, Right Hippocampus (8.4410e-05) | | Right Hippocampus, Right Inferior Temporal Gyrus, anterior division (6.03183e-05) |
| Left Planum Temporale', 'Right Frontal Medial Cortex (1.23262e-06) | Right Juxtapositional Lobule Cortex, Right Parahippocampal Gyrus, (8.9028e-05) | | Right Inferior Frontal Gyrus, pars triangularis, Left Lateral Ventrical (6.7135e-05) |

| | | | |
|---|---|---|---|
| Left Inferior Temporal Gyrus, 'Right Supracalcarine Cortex (1.6064e-06) | Right Intracalcarine Cortex, Left Supracalcarine Cortex (9.0231e-05) | | Left Frontal Orbital Cortex, Left Cerebral White Matter (6.7244e-05) |
| Left Frontal Pole', 'Left Amygdala (2.15358e-06) | Right Middle Temporal Gyrus, posterior division, Left Accumbens (9.0787e-05) | | Left Hippocampus, Right Inferior Temporal Gyrus, anterior division (6.7823e-05) |
| Right Planum Polare', 'Right Middle Temporal Gyrus,(2.2362e-06) | Right Juxtapositional Lobule CortexRight Caudate (9.08e-05) | | Right Inferior Frontal Gyrus, pars opercularis, Right Cuneal Cortex (7.1048e-05) |
| Left Supramarginal Gyrus, anterior division', 'Left Precuneous Cortex (2.46274e-06) | Right Frontal Orbital Cortex, Left Supracalcarine Cortex (9.173e-05) | | Right Temporal Occipital Fusiform Cortex, Right Juxtapositional Lobule Cortex (7.2709e-05) |
| Left Frontal Operculum Cortex', 'Right Caudate (2.51071e-06) | Right Angular Gyrus, Right Accumbens (9.451e-05) | | Right Inferior Frontal Gyrus, pars opercularis, Left Lateral Occipital Cortex, (8.1076e-05) |
| Left Frontal Pole', 'Left Middle Temporal Gyrus, temporooccipital part (2.54912e-06) | Right Hippocampus, Left Temporal Fusiform Cortex, anterior division (9.499e-05) | | Right Paracingulate Gyrus, Right Inferior Temporal Gyrus, anterior division (8.5056e-05) |
| Right Subcallosal Cortex', 'Left Accumbens (2.65357e-06) | Right Lateral Ventricle, Right Accumbens (9.9291e-05) | | Left Frontal Pole, Right Inferior Temporal Gyrus, anterior division (8.5114e-05) |
| Right Lateral Occipital Cortex, , 'Left Precuneous Cortex (2.7160e-06) | Left Pallidum, Left Accumbens (0.0001) | | Right Frontal Operculum Cortex, Right Parahippocampal Gyrus, anterior division (8.7715e- |
| Right Subcallosal Cortex', 'Left Caudate (2.90541e-06) | Left Intracalcarine Cortex, Right Supracalcarine Cortex (0.0001) | | Right Frontal Operculum Cortex, Right Lateral Ventricle (9.064e-05) |
| Left Planum Temporale', 'Right Cingulate Gyrus, anterior division (2.97952e-06) | Right Juxtapositional Lobule Cortex , Right Superior Parietal Lobule (0.0001) | | Left Inferior Frontal Gyrus, pars opercularis, Right Inferior Temporal Gyrus, anterior division (9.0771e- |
| Left Planum Temporale', 'Right Lateral Ventricle (3.56705e-06) | Left Precentral Gyrus, Left Accumbens (0.0001) | | Left Middle Temporal Gyrus, anterior division, Right Inferior Temporal Gyrus, anterior division (9.7773e- |
| Left Frontal Operculum Cortex', 'Right Putamen (3.774e-06) | Right Juxtapositional Lobule Cortex,"Left Heschls Gyrus (includes H1 and H2)") (0.0001) | | Right Accumbens, Right Inferior Temporal Gyrus, anterior division (0.0001) |
| | Left Thalamus, Right Caudate (0.0001) | | Right Inferior Frontal Gyrus, pars triangularis, Left Lingual Gyrus (0.0001) |
| | Right Juxtapositional Lobule Cortex (formerly Supplementary Motor Cortex), Right Inferior Frontal Gyrus, pars triangularis (0.0001) | | Right Inferior Frontal Gyrus, pars opercularis, Left Cingulate Gyrus, posterior division (0.0001) |
| | Left Lateral Occipital Cortex, inferior division, Left Planum Temporale (0.0001) | | Right Inferior Frontal Gyrus, pars triangularis, Right Parahippocampal Gyrus, anterior division (0.0001) |
| | Left Temporal Occipital Fusiform Cortex, Right Paracingulate Gyrus (0.0001) | | Right Inferior Frontal Gyrus, pars triangularis, Left Parahippocampal Gyrus,(0.0001) |
| | Right Juxtapositional Lobule Cortex,Right Planum Temporale (0.0001) | | Right Cerebral White Matter, Right Juxtapositional Lobule Cortex (formerly Supplementary Motor |
| | Right Juxtapositional Lobule Cortex Left Superior Temporal Gyrus, posterior division (0.0001) | | Left Parahippocampal Gyrus, posterior division, Right Inferior Temporal Gyrus, anterior division (0.0001) |
| | Left Juxtapositional Lobule Cortex Right Insular Cortex (0.0001) | | Right Amygdala, Left Cerebral White Matter (0.0001) |
| | Right Angular Gyrus, Right Hippocampus (0.0001) | | Left Cingulate Gyrus, anterior division, Left Cingulate Gyrus, posterior division (0.0001) |
| | Right Subcallosal Cortex, Left Supramarginal Gyrus, anterior division (0.0001) | | Left Cerebral White Matter, Right Juxtapositional Lobule Cortex (formerly Supplementary Motor |
| | Left Temporal Occipital Fusiform Cortex, Left Caudate (0.0001) | | Right Frontal Orbital Cortex, Right Inferior Temporal Gyrus, anterior division (0.0001) |
| | Left Temporal Occipital Fusiform Cortex, Left Superior Temporal Gyrus,(0.0001) | | Right Inferior Frontal Gyrus, pars triangularis, Right Lateral Ventricle (0.0001) |
| | Right Juxtapositional Lobule CortexLeft Parietal Operculum Cortex (0.0001) | | Right Cingulate Gyrus, posterior division, Right Inferior Temporal Gyrus, anterior division (0.0001) |
| | Right Frontal Orbital Cortex, Left Temporal Fusiform Cortex, anterior division (0.0001) | | Right Inferior Frontal Gyrus, pars triangularis, Right Subcallosal Cortex (0.0001) |
| | Right Accumbens, Left Temporal Fusiform Cortex, anterior division (0.0001) | | Right Inferior Frontal Gyrus, pars opercularis, Right Temporal Occipital Fusiform Cortex (0.0001) |
| | Left Juxtapositional Lobule Cortex (formerly Supplementary Motor Cortex), Right Frontal | | Right Inferior Frontal Gyrus, pars triangularis, Right Cuneal Cortex (0.0001) |
| | Right Caudate, Right Inferior Temporal Gyrus, temporooccipital part (0.0001) | | Left Inferior Frontal Gyrus, pars opercularis, Left Cerebral White Matter (0.0001) |
| | Right Hippocampus, Left Accumbens (0.0001) | | Right Frontal Pole, Right Temporal Fusiform Cortex, anterior division (0.0001) |
| | Right Middle Temporal Gyrus, anterior division, Left Accumbens (0.0001) | | Right Cuneal Cortex, Right Juxtapositional Lobule (0.0001) |
| | Right Middle Temporal Gyrus, temporooccipital part, Left Accumbens (0.0002) | | Right Inferior Frontal Gyrus, pars triangularis, Left Hippocampus (0.0001) |
| | Right Temporal Pole, Left Accumbens (0.0002) | | Right Temporal Pole, Temporal Gyrus, anterior division (0.0001) |
| | Right Inferior Temporal Gyrus, temporooccipital part, Left Planum Temporale (0.0002) | | Right Inferior Frontal Gyrus, pars opercularis, Left Lingual Gyrus (0.0001) |
| | Right Juxtapositional Lobule Cortex,Left Caudate (0.0002) | | Right Amygdala, Right Cerebral White Matter (0.0001) |
| | Right Subcallosal Cortex, Left Accumbens (0.0002) | | Right Cingulate Gyrus, anterior division, Left Supracalcarine Cortex (0.0001) |
| | Right Lateral Ventricle, Left Pallidum (0.0002) | | Left Inferior Frontal Gyrus, pars opercularis, Left Lateral Occipital Cortex(0.0001) |
| | Right Lateral Ventricle, Right Hippocampus (0.0002) | | Right Juxtapositional Lobule Cortex, Right Cuneal Cortex (0.0001) |
| | Left Juxtapositional Lobule Cortex (formerly Supplementary Motor Cortex), Right Putamen | | Right Frontal Operculum Cortex, Left Cerebral White Matter (0.0002) |
| | Left Juxtapositional Lobule Cortex,Right Middle Frontal Gyrus (0.0002) | | Left Cingulate Gyrus, anterior division, Right Inferior Temporal Gyrus(0.0002) |
| | Right Juxtapositional Lobule Cortex) Right Supracalcarine Cortex (0.0003) | | Right Central Opercular Cortex, Right Inferior Temporal Gyrus, anterior division (0.0002) |
| | | | Right Cingulate Gyrus, anterior division, Right Cuneal Cortex (0.0002) |

| | | | |
|---|---|---|---|
| | | | Left Thalamus, Right Inferior Temporal Gyrus, anterior division (0.0002) |
| | | | Right Lateral Ventricle, Right Inferior Temporal Gyrus, anterior division (0.0002) |
| | | | Right Frontal Operculum Cortex, Right Subcallosal Cortex (0.0002) |
| | | | Right Paracingulate Gyrus, Left Intracalcarine Cortex (0.0002) |
| | | | Right Inferior Frontal Gyrus, pars triangularis, Right Occipital Pole (0.0002) |
| | | | Right Inferior Frontal Gyrus, pars triangularis, Left Caudate (0.0002) |
| | | | Left Paracingulate Gyrus, Right Cuneal Cortex (0.0002) |
| | | | Left Inferior Frontal Gyrus, pars opercularis, Right Cuneal Cortex (0.0002) |
| | | | Right Middle Temporal Gyrus, posterior division, Left Cerebral White Matter (0.0002) |

<u>Emotional reactivity task significant connections for PTSD prediction at T2</u>

*Format: region A, region B (p-value); sorted by significance.*

| Our Model | Ours E is the identity | Deep fMRI | Ours + GCN |
|---|---|---|---|
| Left Middle Temporal Gyrus, temporooccipital part', 'Left Inferior Temporal Gyrus, posterior division (3.5931e-07) | None | None | None |
| Right Temporal Pole', 'Left Inferior Temporal Gyrus, posterior division (1.03927e-06) | | | |
| Left Precuneous Cortex', 'Left Frontal Medial Cortex (1.23884e-06) | | | |
| Right Temporal Pole', 'Right Putamen (5.2068e-06) | | | |
| Right Cerebral Cortex ', 'Right Superior Temporal Gyrus, anterior division (9.1810e-06) | | | |
| Right Middle Frontal Gyrus', 'Left Middle Frontal Gyrus (1.68810e-05) | | | |

<u>Emotional reactivity task significant connections for PTSD prediction at T3</u>

*Format: region A, region B (p-value); sorted by significance.*

| Our Model | Ours E is the identity | Deep fMRI | Ours + GCN |
|---|---|---|---|
| Right Lateral Occipital Cortex, inferior division', 'Right Caudate (1.48768e-06) | Left Juxtapositional Lobule Cortex (formerly Supplementary *Motor* Cortex), Right Middle Temporal Gyrus, posterior division (1.0899e-06) | Left Lateral Occipital Cortex, superior division, Right Supracalcarine Cortex (4.13626e-06) | None |
| Right Accumbens', 'Right Lingual Gyrus (1.63612e-06) | Right Juxtapositional Lobule Cortex, Right Frontal Medial Cortex (3.0679e-06) | Right Supracalcarine Cortex, Left Lateral Occipital Cortex, (4.13626-06) | |
| Right Temporal Fusiform Cortex, posterior division', 'Right Accumbens (1.07673e-05) | Right Precentral Gyrus, Right Inferior Frontal Gyrus, pars triangularis (4.920e-06) | Right Occipital Fusiform Gyrus, Right Supracalcarine Cortex (8.7078e-06) | |
| Right Lateral Occipital Cortex, inferior division', 'Right Lateral Occipital Cortex, superior division (1.10220e-05) | Left Postcentral Gyrus, Right Middle Temporal Gyrus, posterior division (7.431e-06) | Right Supracalcarine Cortex, Right Occipital Fusiform Gyrus (8.7078e-06) | |
| Right Lateral Occipital Cortex, inferior division', 'Right Frontal Medial Cortex (1.29057e-05) | Left Thalamus, Right Inferior Frontal Gyrus, pars triangularis (8.795e-06) | Right Supramarginal Gyrus, anterior division, Left Superior Parietal Lobule (2.1768e-05) | |
| Right Supramarginal Gyrus, anterior division', 'Left Supramarginal Gyrus, posterior division (1.38638e-05) | Right Temporal Pole, Right Middle Temporal Gyrus, posterior division (9.73e-06) | Left Superior Parietal Lobule, Right Supramarginal Gyrus, (2.1768e-05) | |
| Right Lingual Gyrus', 'Right Lateral Ventricle (1.48003e-05) | Left Temporal Fusiform Cortex, anterior division, Right Inferior Frontal Gyrus, pars triangularis (1.0871e-05) | | |
| Right Supramarginal Gyrus, anterior division', 'Left Inferior Frontal Gyrus, pars opercularis (1.7119e-05) | Right Juxtapositional Lobule Cortex (formerly Supplementary Motor Cortex), Right Inferior Frontal Gyrus, pars triangularis (1.4628e-05) | | |
| Right Temporal Occipital Fusiform Cortex', 'Left Parahippocampal Gyrus, anterior division (1.7691e-05) | Right Supramarginal Gyrus, anterior division, Right Inferior Frontal Gyrus, pars triangularis (1.6131e-05) | | |
| Right Lateral Occipital Cortex, inferior division', 'Right Inferior Frontal Gyrus, pars opercularis (1.83107e-05) | Right Cingulate Gyrus, posterior division, Right Inferior Frontal Gyrus, pars triangularis (1.652e-05) | | |
| Right Lateral Occipital Cortex, inferior division', 'Left Cerebral White Matter (1.83925e-05) | Left Postcentral Gyrus, Right Temporal Fusiform Cortex, anterior division (1.6717e-05) | | |
| Right Occipital Pole', 'Left Superior Parietal Lobule (1.87835e-05) | Left Juxtapositional Lobule Cortex ,Right Frontal Orbital Cortex (1.7255e-05) | | |
| Right Supramarginal Gyrus, anterior division', 'Left Supracalcarine Cortex (1.92644e-05) | Left Temporal Fusiform Cortex, anterior division, Right Middle Temporal Gyrus, posterior division (1.830e-05) | | |
| Right Parahippocampal Gyrus, posterior division', 'Left Temporal Fusiform Cortex, anterior division (1.9603e-05) | Right Postcentral Gyrus, Right Frontal Medial Cortex (1.8988e-05) | | |
| Right Lingual Gyrus', 'Right Thalamus (1.97126e-05) | Right Juxtapositional Lobule Cortex,Right Middle Temporal Gyrus, posterior division (2.2525e-05) | | |
| Right Lateral Occipital Cortex, 'Right Superior Frontal Gyrus (2.7035e-05) | Right Postcentral Gyrus, Right Middle Temporal Gyrus, (2.332e-05) | | |
| Right Lateral Occipital Cortex, 'Right Cerebral White Matter (2.82953e-05) | Right Temporal Occipital Fusiform Cortex, Left Postcentral Gyrus (2.587e-05) | | |
| Right Temporal Fusiform Cortex, 'Right Caudate (3.27288e-05) | Left Temporal Fusiform Cortex, Right Temporal Fusiform Cortex,(2.8962e-05) | | |

| | | | |
|---|---|---|---|
| Right Parahippocampal Gyrus, , 'Right Lingual Gyrus (3.45765e-05) | Left Postcentral Gyrus, Right Temporal Occipital Fusiform Cortex (3.27e-05) | | |
| Right Supramarginal Gyrus, 'Left Intracalcarine Cortex (3.6986e-05) | Left Occipital Pole, Left Supramarginal Gyrus(3.868e-05) | | |
| Right Occipital Pole', 'Right Caudate (3.93e-05) | Left Thalamus, Right Frontal Medial Cortex (4.473e-05) | | |
| Right Lateral Occipital Cortex, , 'Right Middle Frontal Gyrus (3.9504e-05) | Right Postcentral Gyrus, Right Inferior Frontal Gyrus, pars triangularis (4.8321e-05) | | |
| Right Occipital Pole', 'Left Thalamus (4.34320e-05) | Right Precuneous Cortex, Right Inferior Frontal Gyrus, pars triangularis (5.060e-05) | | |
| Right Lingual Gyrus', 'Right Superior Parietal Lobule (4.8907e-05) | Left Postcentral Gyrus, Brain-Stem (5.3496e-05) | | |
| Right Temporal Fusiform Cortex,Right Cerebral White Matter (5.1405e-05) | Left Occipital Pole, Left Inferior Frontal Gyrus, pars opercularis (7.3342e-05) | | |
| Left Inferior Frontal Gyrus, pars opercularis', 'Right Lateral Ventricle (5.26224e-05) | Left Postcentral Gyrus, Right Inferior Frontal Gyrus, pars triangularis (7.6026e-05) | | |
| ("Right Heschl's Gyrus, Right Accumbens (5.29216e-05) | Left Precentral Gyrus, Right Inferior Frontal Gyrus, pars triangularis (8.6695e-05) | | |
| Left Pallidum', 'Right Parietal Operculum Cortex (6.2781e-05) | Right Cingulate Gyrus, anterior division, Right Inferior Frontal Gyrus, pars triangularis (8.6802e-05) | | |
| ("Right Heschl's Gyrus 'Left Superior Parietal Lobule (6.3317e-05) | Left Precentral Gyrus, Right Frontal Medial Cortex (9.385e-05) | | |
| Right Lateral Occipital Cortex, inferior division', 'Left Thalamus (6.6271e-05) | | | |
| Right Supramarginal Gyrus, 'Right Supracalcarine Cortex (6.8776e-05) | | | |
| Left Temporal Occipital Fusiform Cortex, Right Superior Frontal Gyrus (6.986e-05) | | | |
| Right Temporal Occipital Fusiform Cortex', 'Brain-Stem (7.2768e-05) | | | |
| Right Inferior Temporal Gyrus, 'Left Superior Temporal Gyrus, (7.6271e-05) | | | |
| Right Occipital Pole', 'Right Middle Frontal Gyrus (7.9067e-05) | | | |
| Left Hippocampus', 'Right Lateral Ventricle (8.4812e-05) | | | |
| Right Lingual Gyrus', 'Right Caudate (8.5946e-05) | | | |
| Left Putamen', 'Right Middle Frontal Gyrus (8.8332e-05) | | | |
| Brain-Stem', 'Right Amygdala (0.0001) | | | |
| Left Inferior Frontal Gyrus, 'Left Superior Temporal Gyrus, (0.0001) | | | |
| Left Middle Temporal Gyrus, , 'Right Lateral Occipital Cortex, (0.0001) | | | |
| Right Lateral Occipital Cortex, inferior division', 'Left Hippocampus (0.0001) | | | |
| ("Right Heschl's Gyrus,'Right Superior Parietal Lobule (0.0001) | | | |
| Left Planum Polare', 'Left Superior Temporal Gyrus(0.0001) | | | |
| Left Middle Temporal Gyrus, posterior division', 'Left Superior Parietal Lobule (0.0001)Right Occipital Pole', 'Right Accumbens (0.0001) | | | |
| Left Frontal Medial Cortex', 'Left Superior Parietal Lobule (0.0001) | | | |
| Left Frontal Orbital Cortex', 'Right Lateral Ventricle (0.0001) | | | |
| Right Lateral Occipital Cortex, 'Left Supramarginal Gyrus, (0.0001) | | | |
| Left Occipital Fusiform Gyrus', 'Left Superior Temporal Gyrus, (0.0001) | | | |
| Right Inferior Temporal Gyrus, , 'Left Parahippocampal Gyrus, (0.0001) | | | |
| Brain-Stem', 'Left Parahippocampal Gyrus, posterior division (0.0001) | | | |
| Left Putamen', 'Left Superior Temporal Gyrus, anterior division (0.0001) | | | |
| Left Pallidum', 'Left Middle Frontal Gyrus (0.0001) | | | |
| Right Occipital Pole', 'Left Lateral Ventrical (0.0001) | | | |
| Left Temporal Occipital Fusiform Cortex', 'Right Lateral Ventricle (0.0001) | | | |
| Left Hippocampus', 'Right Accumbens (0.0001) | | | |
| Right Temporal Fusiform Cortex, 'Right Lateral Ventricle (0.0001) | | | |
| Left Middle Temporal Gyrus, , 'Right Occipital Pole (0.0001) | | | |
| Right Lateral Occipital Cortex, 'Left Inferior Frontal Gyrus(0.0001) | | | |
| Left Cerebral Cortex ', 'Right Occipital Pole (0.0001) | | | |
| Left Lateral Ventrical', 'Left Superior Temporal Gyrus(0.0001) | | | |
| Right Middle Frontal Gyrus', 'Left Superior Temporal Gyrus, (0.0002) | | | |
| Left Middle Frontal Gyrus', 'Right Accumbens (0.0002) | | | |
| Right Lateral Occipital Cortex, , 'Left Parahippocampal Gyrus, (0.0002) | | | |
| Left Parahippocampal Gyrus, 'Right Supramarginal Gyrus, (0.0002) | | | |
| Right Temporal Fusiform Cortex, , 'Left Superior Temporal Gyrus, (0.0002) | | | |
| Right Middle Temporal Gyrus, 'Right Cerebral White Matter (0.0002) | | | |
| Right Temporal Fusiform Cortex, 'Right Lateral Occipital Cortex, (0.0002) | | | |
| Left Occipital Fusiform Gyrus', 'Brain-Stem (0.0002) | | | |
| Right Occipital Pole', 'Left Superior Temporal Gyrus(0.0002) | | | |
| Right Lateral Occipital Cortex, 'Left Superior Temporal Gyrus, (0.0002) | | | |
| Right Occipital Pole', 'Right Lateral Ventricle (0.0002) | | | |
| Right Lingual Gyrus', 'Left Lateral Ventrical (0.0002) | | | |
| Left Inferior Frontal Gyrus, pars opercularis', 'Left Lateral Ventrical (0.0002) | | | |
| Left Subcallosal Cortex', 'Right Accumbens (0.0002) | | | |
| Left Temporal Occipital Fusiform Cortex', 'Right Accumbens (0.0002) | | | |
| Right Lateral Occipital Cortex'Left Lateral Ventrical (0.0002) | | | |
| Right Lateral Occipital Cortex,, 'Right Superior Temporal Gyrus, (0.0002) | | | |
| Right Lateral Occipital Cortex,'Right Middle Temporal Gyrus (0.0002) | | | |
| Right Temporal Occipital Fusiform Cortex,Right Parahippocampal Gyrus, (0.0002) | | | |
| Right Lateral Occipital Cortex, inferior division', 'Right Pallidum (0.0003) | | | |
| Right Temporal Fusiform Cortex, 'Left Temporal Fusiform Cortex, (0.0003) | | | |

Reward sensitivity task significant connections for PTSD prediction at T1

*Format: region A, region B (p-value); sorted by significance.*

| Our Model | Ours E is the identity | Deep fMRI | Ours + GCN |
|---|---|---|---|
| Right Superior Temporal Gyrus, Right Inferior Frontal Gyrus (1.16748e-06) | Left Cerebral Cortex ', 'Left Frontal Operculum Cortex (2.209e-07) | None | Left Insular Cortex, Right Superior Temporal Gyrus, (2.151e-06) |
| Left Occipital Pole, Left Lateral Occipital Cortex, superior division (1.4198e-06) | Left Inferior Temporal Gyrus, 'Right Supramarginal Gyrus (3.6932e-07) | | Left Caudate ,Left Planum Temporale (2.207e-06) |
| Right Occipital Fusiform Gyrus, Left Lateral Occipital Cortex, superior division (2.0565e-06) | Left Inferior Temporal Gyrus, 'Left Frontal Operculum Cortex(1.806e-06) | | Left Insular Cortex', 'Right Supracalcarine Cortex' (8.757e-06) |
| Right Occipital Pole, Left Lateral Occipital Cortex, superior division (2.61443e-06) | Left Cerebral White Matter', 'Left Frontal Operculum Cortex' (4.22e-06) | | |
| Right Temporal Fusiform Cortex, anterior division, Left Lateral Occipital Cortex (3.13511e-06) | Left Inferior Temporal Gyrus, 'Left Insular Cortex (6.131e-06) | | |
| Left Caudate, Right Lateral Occipital Cortex, superior division (3.3559e-06) | | | |
| Left Occipital Fusiform Gyrus, Left Lateral Occipital Cortex, superior division (3.4897e-06) | | | |
| Left Putamen, Left Lateral Occipital Cortex, superior division (3.7469e-06) | | | |
| Left Juxtapositional Lobule Cortex Left Lateral Occipital Cortex, superior division (4.307238e-06) | | | |
| Left Inferior Temporal Gyrus, anterior division, Left Lateral Occipital Cortex,(4.4433e-06) | | | |
| Right Caudate, Left Lateral Occipital Cortex, superior division (4.604251e-06) | | | |
| Right Inferior Frontal Gyrus, pars opercularis, Left Lateral Occipital Cortex,(4.7130e-06) | | | |
| Left Superior Parietal Lobule, Left Lateral Occipital Cortex, superior division (6.2587e-06) | | | |
| Left Superior Parietal Lobule, Right Superior Temporal Gyrus, anterior division (6.3537e-06) | | | |
| Right Frontal Pole, Right Lingual Gyrus (6.4966e-06) | | | |
| Right Cingulate Gyrus, posterior division, Left Superior Parietal Lobule (7.10706e-06) | | | |
| Right Supracalcarine Cortex, Left Lateral Occipital Cortex, superior division (7.310100e-06) | | | |
| Right Occipital Pole, Right Cingulate Gyrus, anterior division (7.5103e-06) | | | |
| Right Lateral Occipital Cortex, inferior division, Left Lateral Occipital Cortex, (7.5385e-06) | | | |
| Left Middle Frontal Gyrus, Left Lateral Occipital Cortex, superior division (7.71908e-06) | | | |
| Left Pallidum, Right Lateral Occipital Cortex, superior division (7.7701e-06) | | | |
| Right Inferior Temporal Gyrus, posterior division, Right Lingual Gyrus 8.90155e-06) | | | |
| Left Superior Parietal Lobule, Right Inferior Frontal Gyrus, pars opercularis 9.012639e-06) | | | |
| Left Angular Gyrus, Left Lateral Occipital Cortex, superior division 9.0350e-06) | | | |
| Right Middle Temporal Gyrus, posterior division, Left Lateral Occipital Cortex, 9.21785e-06) | | | |
| Left Inferior Temporal Gyrus, posterior division, Left Lateral Occipital Cortex, 9.34003e-06) | | | |
| Left Angular Gyrus, Right Lateral Occipital Cortex, superior division 9.5196e-06) | | | |
| Left Middle Temporal Gyrus, temporooccipital part, Right Middle Frontal Gyrus 9.6102e-06) | | | |
| Left Postcentral Gyrus, Left Lateral Occipital Cortex, superior division (1.0245e-05) | | | |
| Left Temporal Pole, Right Lateral Occipital Cortex, superior division (1.0475e-05) | | | |
| Left Middle Temporal Gyrus, temporooccipital part, Left Lateral Occipital Cortex(1.0755e-05) | | | |
| Left Supramarginal Gyrus, anterior division, Left Lateral Occipital Cortex,(1.1160e-05) | | | |
| Left Precentral Gyrus, Right Inferior Frontal Gyrus, pars opercularis (1.1360e-05) | | | |
| Left Occipital Fusiform Gyrus, Right Middle Frontal Gyrus (1.1654e-05) | | | |
| Left Inferior Temporal Gyrus, temporooccipital part, Left Lateral Occipital Cortex, (1.1697e-05) | | | |
| Right Planum Polare, Left Lateral Occipital Cortex, superior division (1.2396e-05) | | | |
| Left Cingulate Gyrus, posterior division, Right Lateral Occipital Cortex,(1.2881e-05) | | | |
| Right Precentral Gyrus, Left Lateral Occipital Cortex, superior division (1.3053e-05) | | | |
| Right Temporal Occipital Fusiform Cortex, Left Lateral Occipital Cortex,(1.3225e-05) | | | |
| Left Inferior Frontal Gyrus, pars opercularis, Left Lateral Occipital Cortex, (1.4246e-05) | | | |
| Right Subcallosal Cortex, "Left Heschls Gyrus (includes H1 and H2)") (1.4303e-05) | | | |
| Left Occipital Fusiform Gyrus, Right Precuneous Cortex (1.5287e-05) | | | |
| Right Cingulate Gyrus, anterior division, Left Lateral Occipital Cortex, (1.5297e-05) | | | |
| Right Thalamus, Left Lateral Occipital Cortex, superior division (1.5440e-05) | | | |
| Right Superior Parietal Lobule, Left Lateral Occipital Cortex, superior division (1.6594e-05) | | | |
| Right Insular Cortex, Right Lateral Occipital Cortex, superior division (1.695e-05) | | | |
| Right Planum Polare, Right Inferior Frontal Gyrus, pars opercularis (1.7006e-05) | | | |
| Right Pallidum, Left Lateral Occipital Cortex, superior division (1.7204e-05) | | | |
| Right Supramarginal Gyrus, anterior division, Left Lateral Occipital Cortex, (1.7483e-05) | | | |
| Right Occipital Pole, Right Inferior Frontal Gyrus, pars opercularis (1.7562e-05) | | | |
| Left Juxtapositional Lobule Cortex), Left Angular Gyrus (1.7624e-05) | | | |
| ("Right Heschls Gyrus (includes H1 and H2)", Right Lateral Occipital Cortex, (1.8039e-05) | | | |
| Right Inferior Frontal Gyrus, pars triangularis, Right Lateral Occipital Cortex(1.8132e-05) | | | |
| Right Cingulate Gyrus, anterior division, Right Lateral Occipital Cortex,(1.8339e-05) | | | |
| Right Middle Temporal Gyrus, anterior division, Left Lateral Occipital Cortex,(1.8735e-05) | | | |
| Left Parietal Operculum Cortex, Right Lateral Occipital Cortex, superior division (1.8971e-05) | | | |
| Left Superior Temporal Gyrus, anterior division, Left Lateral Occipital Cortex, | | | |
| Left Juxtapositional Lobule Cortex Right Middle Frontal Gyrus (1.9374e-05) | | | |
| Right Parahippocampal Gyrus, posterior division, Left Lateral Occipital Cortex,1.9993e-05) | | | |
| Right Temporal Fusiform Cortex, anterior division, Right Middle Frontal Gyrus (2.0458e-05) | | | |
| Right Cingulate Gyrus, posterior division, Left Lateral Occipital Cortex,(2.0495e-05) | | | |
| Left Temporal Occipital Fusiform Cortex, Left Lateral Occipital Cortex,(2.0496e-05) | | | |
| Left Insular Cortex, Right Lateral Occipital Cortex, superior division (2.108e-05) | | | |
| Right Accumbens, Left Lateral Occipital Cortex, superior division (2.1300e-05) | | | |
| Right Caudate, Right Middle Frontal Gyrus (2.1403e-05) | | | |
| Right Occipital Pole, Right Inferior Frontal Gyrus, pars triangularis (2.3150e-05) | | | |
| Left Postcentral Gyrus, Right Inferior Frontal Gyrus, pars triangularis (2.349e-05) | | | |
| Right Cingulate Gyrus, posterior division, Right Inferior Frontal Gyrus,(2.3734e-05) | | | |
| Left Hippocampus, Left Lateral Occipital Cortex, superior division (2.3888e-05) | | | |
| Right Superior Temporal Gyrus, anterior division, Left Superior Parietal Lobule (2.3894e-05) | | | |
| Left Superior Frontal Gyrus, Left Lateral Occipital Cortex, superior division (2.40926e-05) | | | |
| Right Superior Temporal Gyrus, anterior division, Left Lateral Occipital Cortex (2.4482e-05) | | | |
| Right Intracalcarine Cortex, Right Lateral Occipital Cortex, superior division (2.47087e-05) | | | |
| Left Paracingulate Gyrus, Right Lingual Gyrus (2.4903e-05) | | | |

| | | | |
|---|---|---|---|
| Left Precuneous Cortex, Left Lateral Occipital Cortex, superior division (2.5419e-05) | | | |
| Right Frontal Orbital Cortex, Right Lateral Occipital Cortex, superior division (2.5702e-05) | | | |
| Right Parahippocampal Gyrus, anterior division, Left Lateral Occipital Cortex,(2.5884e-05) | | | |
| Right Frontal Orbital Cortex, Left Lateral Occipital Cortex, superior division (2.6052e-05) | | | |
| Left Frontal Medial Cortex, Left Lateral Occipital Cortex, superior division (2.71276e-05) | | | |
| Right Temporal Pole, Left Lateral Occipital Cortex, superior division (2.7436e-05) | | | |
| Right Frontal Pole, Right Hippocampus (2.7459e-05) | | | |
| Left Superior Temporal Gyrus, posterior division, Right Lateral Occipital Cortex,(2.7614e-05) | | | |
| Right Superior Frontal Gyrus, Left Lateral Occipital Cortex, superior division (2.8468e-05) | | | |
| Left Frontal Pole, Left Accumbens (2.8647e-05) | | | |
| Right Superior Temporal Gyrus, anterior division, Right Lateral Occipital Cortex,(2.8748e-05) | | | |
| Left Juxtapositional Lobule Cortex (formerly Supplementary Motor Cortex), Right Lateral Occipital Cortex, superior division (2.937e-05) | | | |
| Right Temporal Occipital Fusiform Cortex, Right Inferior Frontal Gyrus(3.070e-05) | | | |
| Right Middle Frontal Gyrus, Left Lateral Occipital Cortex, superior division (3.1157e-05) | | | |
| Left Lingual Gyrus, Left Angular Gyrus (3.1319e-05) | | | |
| Right Amygdala, Right Lateral Occipital Cortex, superior division (3.1723e-05) | | | |
| Right Parietal Operculum Cortex, Right Lateral Occipital Cortex, superior division (3.1809e-05) | | | |
| Left Parahippocampal Gyrus, anterior division, Right Lateral Occipital Corte (3.2978e-05) | | | |
| Left Amygdala, Right Lateral Occipital Cortex, superior division (3.3819e-05) | | | |
| Left Middle Temporal Gyrus, anterior division, Right Lateral Occipital Cortex(3.417e-05) | | | |
| Right Caudate, Left Angular Gyrus (3.5193e-05) | | | |
| Right Pallidum, Left Superior Parietal Lobule (3.5351e-05) | | | |

Reward sensitivity task  significant connections for PTSD prediction at T2

*Format: region A, region B (p-value); sorted by significance.*

| Our Model | Ours E is the identity | Deep fMRI | Ours + GCN |
|---|---|---|---|
| Right Lingual Gyrus, Right Cuneal Cortex (2.7380e-10) | None | None | None |
| Left Caudate, Left Supracalcarine Cortex (6.4652e-10) | | | |
| Left Cingulate Gyrus, posterior division, Right Lateral Occipital Cortex, superior division (6.5231e-10) | | | |
| Left Cingulate Gyrus, posterior division, Left Lateral Occipital Cortex, superior division (6.5409e-10) | | | |
| Left Cingulate Gyrus, posterior division, Right Occipital Pole 8.7359e-10) | | | |
| Left Cingulate Gyrus, posterior division, Right Cuneal Cortex 9.8927e-10) | | | |
| Right Lingual Gyrus, Left Lateral Occipital Cortex, superior division (1.82917e-09) | | | |
| Right Planum Temporale, Left Supracalcarine Cortex (2.63502e-09) | | | |
| Left Caudate, Left Intracalcarine Cortex (2.69236e-09) | | | |
| Right Lingual Gyrus, Left Middle Temporal Gyrus, posterior division (3.7914e-09) | | | |
| Left Accumbens, Left Lateral Occipital Cortex, superior division (4.4327e-09) | | | |
| Right Frontal Operculum Cortex, Right Intracalcarine Cortex (5.11072e-09) | | | |
| Right Superior Temporal Gyrus, posterior division, Left Supracalcarine Cortex (5.3201e-09) | | | |
| Left Cingulate Gyrus, posterior division, Left Cerebral Cortex  (5.3275e-09) | | | |
| Left Cingulate Gyrus, posterior division, Left Hippocampus (5.53598e-09) | | | |
| Right Cingulate Gyrus, posterior division, Left Lateral Occipital Cortex, superior division (5.8858e-09) | | | |
| Right Cingulate Gyrus, posterior division, Right Lateral Occipital Cortex, superior division (6.7133e-09) | | | |
| Left Paracingulate Gyrus, Left Lateral Occipital Cortex, superior division (6.7961e-09) | | | |
| Right Inferior Frontal Gyrus, pars opercularis, Left Lateral Occipital Cortex, superior division (7.6166e-09) | | | |
| Left Caudate, Right Intracalcarine Cortex (7.6259e-09) | | | |
| Right Planum Temporale, Right Intracalcarine Cortex (7.8626e-09) | | | |
| Left Cingulate Gyrus, posterior division, Left Intracalcarine Cortex (7.9887e-09) | | | |
| Left Middle Frontal Gyrus, Right Cuneal Cortex 8.0549e-09) | | | |
| Left Accumbens, Left Hippocampus 8.5171e-09) | | | |
| Left Amygdala, Left Lateral Occipital Cortex, superior division 8.8823e-09) | | | |
| Left Cingulate Gyrus, posterior division, Right Superior Frontal Gyrus (1.2504e-08) | | | |
| Left Accumbens, Left Putamen (1.2854e-08) | | | |
| Left Accumbens, Right Occipital Pole (1.4741e-08) | | | |
| Right Parahippocampal Gyrus, anterior division, Left Lateral Occipital Cortex, superior division (1.7728e-08) | | | |
| Left Temporal Pole, Left Lateral Occipital Cortex, superior division (1.7838e-08) | | | |
| Right Cerebral Cortex , Right Cuneal Cortex (1.8804e-08) | | | |
| Left Accumbens, Right Lateral Occipital Cortex, superior division (1.93050e-08) | | | |
| Left Cingulate Gyrus, posterior division, Left Middle Temporal Gyrus, temporooccipital part (1.9884e-08) | | | |
| Left Superior Temporal Gyrus, posterior division, Left Supracalcarine Cortex (2.0162e-08) | | | |
| Right Occipital Pole, Left Supracalcarine Cortex (2.06537e-08) | | | |
| Left Lingual Gyrus, Right Cuneal Cortex (2.0940e-08) | | | |
| Left Cingulate Gyrus, posterior division, Left Temporal Pole (2.1649e-08) | | | |
| Left Accumbens, Right Pallidum (2.20497e-08) | | | |
| Left Cingulate Gyrus, posterior division, Right Intracalcarine Cortex (2.24885e-08) | | | |
| Left Accumbens, Right Cuneal Cortex (2.2984e-08) | | | |
| Right Superior Temporal Gyrus, posterior division, Right Intracalcarine Cortex (2.3541e-08) | | | |
| Right Lateral Ventricle, Left Lateral Occipital Cortex, superior division (2.5551e-08) | | | |
| Left Precuneous Cortex, Left Lateral Occipital Cortex, superior division (2.63511e-08) | | | |
| Left Caudate, Left Planum Temporale (3.0488e-08) | | | |
| Left Accumbens, Right Thalamus (3.1331e-08) | | | |
| Left Cingulate Gyrus, posterior division, Right Inferior Temporal Gyrus, posterior division (3.7306e-08) | | | |
| Right Cerebral White Matter, Left Lateral Ventrical (4.1035e-08) | | | |
| Left Cingulate Gyrus, posterior division, Right Cerebral White Matter (4.3653e-08) | | | |
| Right Cerebral Cortex , Left Lateral Occipital Cortex, superior division (5.1932e-08) | | | |
| Right Precentral Gyrus, Left Lateral Occipital Cortex, superior division (5.2013e-08) | | | |

| | | | |
|---|---|---|---|
| Left Accumbens, Right Temporal Fusiform Cortex, posterior division (5.6724e-08) | | | |
| Right Precuneous Cortex, Left Lateral Occipital Cortex, superior division (6.2449e-08) | | | |
| Left Cingulate Gyrus, posterior division, Left Precentral Gyrus (6.35146e-08) | | | |
| Left Superior Temporal Gyrus, posterior division, Right Intracalcarine Cortex (6.63609e-08) | | | |
| Right Frontal Pole, Left Intracalcarine Cortex (6.89949e-08) | | | |
| Right Inferior Temporal Gyrus, temporooccipital part, Right Lateral Occipital Cortex,(7.6326e-08) | | | |
| Right Cingulate Gyrus, posterior division, Left Occipital Pole (7.73125e-08) | | | |
| Left Occipital Pole, Left Supracalcarine Cortex (7.90616e-08) | | | |
| Left Accumbens, Right Parahippocampal Gyrus, anterior division 8.20078e-08) | | | |
| Left Accumbens, Left Inferior Temporal Gyrus, anterior division 8.27238e-08) | | | |
| Left Middle Frontal Gyrus, Left Intracalcarine Cortex 8.30099e-08) | | | |
| Left Accumbens, Left Thalamus 8.60566e-08) | | | |
| Left Accumbens, Left Intracalcarine Cortex 8.63892e-08) | | | |
| Right Lingual Gyrus, Left Intracalcarine Cortex 8.82059e-08) | | | |
| Right Lingual Gyrus, Left Middle Temporal Gyrus, temporooccipital part 9.78995e-08) | | | |
| Right Inferior Temporal Gyrus, temporooccipital part, Left Intracalcarine Cortex (1.0115e-07) | | | |
| Left Cingulate Gyrus, posterior division, Left Precuneous Cortex (1.0334e-07) | | | |
| Left Superior Temporal Gyrus, posterior division, Right Cuneal Cortex (1.0387e-07) | | | |
| Right Frontal Pole, Right Lateral Occipital Cortex, superior division (1.0823e-07) | | | |
| Right Cingulate Gyrus, posterior division, Right Cuneal Cortex (1.0869e-07) | | | |
| Right Inferior Frontal Gyrus, pars triangularis, Left Lateral Occipital Cortex, superior division (1.1097e-07) | | | |
| Left Cingulate Gyrus, posterior division, Left Pallidum (1.1252e-07) | | | |
| Right Cingulate Gyrus, posterior division, Left Intracalcarine Cortex (1.1256e-07) | | | |
| Left Cingulate Gyrus, posterior division, Left Postcentral Gyrus (1.1381e-07) | | | |
| Right Caudate, Right Supracalcarine Cortex (1.1952e-07) | | | |
| Right Cerebral White Matter, Right Lateral Ventricle (1.1968e-07) | | | |
| Right Inferior Temporal Gyrus, temporooccipital part, Right Intracalcarine Cortex (1.3838e-07) | | | |
| Right Precentral Gyrus, Right Cuneal Cortex (1.393e-07) | | | |
| Left Cingulate Gyrus, posterior division, Left Frontal Medial Cortex (1.4033e-07) | | | |
| Right Occipital Pole, Right Intracalcarine Cortex (1.4529e-07) | | | |
| Right Lingual Gyrus, Left Thalamus (1.5742e-07) | | | |
| Right Cingulate Gyrus, posterior division, Right Intracalcarine Cortex (1.6628e-07) | | | |
| Left Occipital Pole, Right Intracalcarine Cortex (1.6710e-07) | | | |
| Left Superior Temporal Gyrus, posterior division, Left Intracalcarine Cortex (1.680e-07) | | | |
| Right Lingual Gyrus, Right Occipital Pole (1.7466e-07) | | | |
| Left Cingulate Gyrus, posterior division, Left Supramarginal Gyrus, posterior division (1.823e-07) | | | |
| Left Accumbens, Right Intracalcarine Cortex (1.8817e-07) | | | |
| Left Cuneal Cortex, Right Cuneal Cortex (1.8968e-07) | | | |
| Left Cingulate Gyrus, posterior division, Left Inferior Frontal Gyrus, pars triangularis (1.9372e-07) | | | |
| Right Lingual Gyrus, Left Cuneal Cortex (1.9677e-07) | | | |
| Left Accumbens, Left Middle Frontal Gyrus (2.020e-07) | | | |
| Left Caudate, Left Superior Temporal Gyrus, posterior division (2.0638e-07) | | | |
| Right Lateral Ventricle, Left Lateral Ventrical (2.093e-07) | | | |
| Left Caudate, Right Occipital Fusiform Gyrus (2.254e-07) | | | |
| Right Middle Frontal Gyrus, Right Intracalcarine Cortex (2.327e-07) | | | |
| Left Temporal Pole, Right Cuneal Cortex (2.430e-07) | | | |
| Left Cingulate Gyrus, posterior division, Right Parietal Operculum Cortex (2.5417e-07) | | | |
| Left Accumbens, Left Parahippocampal Gyrus, posterior division (2.5865e-07) | | | |
| Left Frontal Pole, Left Lateral Occipital Cortex, superior division (2.7416e-07) | | | |
| Right Parahippocampal Gyrus, anterior division, Right Lateral Ventricle (2.9527e-07) | | | |
| Left Parietal Operculum Cortex, Left Lateral Occipital Cortex, superior division (3.0457e-07) | | | |
| Right Cerebral Cortex , Right Lateral Occipital Cortex, superior division (3.1304e-07) | | | |
| Right Occipital Pole, Left Intracalcarine Cortex (3.3655e-07) | | | |
| Right Inferior Temporal Gyrus, temporooccipital part, Left Lateral Occipital Cortex, superior division (3.388e-07) | | | |
| Left Cingulate Gyrus, posterior division, Right Frontal Medial Cortex (3.550e-07) | | | |
| Left Hippocampus, Left Lateral Occipital Cortex, superior division (3.5628e-07) | | | |
| Left Caudate, Left Cuneal Cortex (3.6151e-07) | | | |
| ("Right Heschls Gyrus (includes H1 and H2)", Left Intracalcarine Cortex (3.6898e-07) | | | |
| Left Caudate, Left Middle Temporal Gyrus, posterior division (3.7818e-07) | | | |
| Left Inferior Frontal Gyrus, pars opercularis, Right Inferior Temporal Gyrus, anterior division (3.8389e-07) | | | |
| Left Accumbens, Left Occipital Fusiform Gyrus (3.883e-07) | | | |
| Left Accumbens, Right Frontal Orbital Cortex (4.247e-07) | | | |
| Left Inferior Frontal Gyrus, pars triangularis, Left Frontal Orbital Cortex (4.4040e-07) | | | |
| Right Frontal Medial Cortex, Left Intracalcarine Cortex (4.4495e-07) | | | |
| Left Frontal Pole, Left Intracalcarine Cortex (4.6054e-07) | | | |
| Left Caudate, Left Lateral Occipital Cortex, superior division (4.7209e-07) | | | |
| Left Accumbens, Left Frontal Medial Cortex(4.771e-07) | | | |
| Right Occipital Fusiform Gyrus, Left Supracalcarine Cortex (4.812e-07)) | | | |
| Left Cingulate Gyrus, posterior division, Right Supramarginal Gyrus, anterior division (4.837e-07) | | | |
| Left Accumbens, Left Middle Temporal Gyrus, anterior division (5.02839e-07) | | | |
| Left Cingulate Gyrus, posterior division, Right Frontal Orbital Cortex (5.12596e-07) | | | |
| Left Cingulate Gyrus, posterior division, Left Insular Cortex (5.56745e-07) | | | |
| Right Frontal Pole, Left Lateral Occipital Cortex, superior division (5.65155e-07) | | | |
| ("Left Heschls Gyrus (includes H1 and H2)", Right Intracalcarine Cortex (5.7017e-07) | | | |
| Left Accumbens, "Right Heschls Gyrus (includes H1 and H2)") (5.7063e-07) | | | |
| Right Superior Frontal Gyrus, Right Cuneal Cortex (5.7493e-07) | | | |
| Left Cingulate Gyrus, posterior division, Right Postcentral Gyrus (5.8716e-07) | | | |
| Right Parahippocampal Gyrus, anterior division, Left Lateral Ventrical (5.999e-07) | | | |
| Left Accumbens, Left Precuneous Cortex (6.0619e-07) | | | |
| Right Superior Temporal Gyrus, posterior division, Right Supracalcarine Cortex (6.2113e-07) | | | |
| Left Accumbens, Left Middle Temporal Gyrus, temporooccipital part (6.2628e-07) | | | |
| Left Inferior Temporal Gyrus, posterior division, Right Lateral Ventricle (6.567e-07) | | | |

| | | | |
|---|---|---|---|
| Left Accumbens, "Left Heschls Gyrus (includes H1 and H2)") (6.6700e-07) | | | |
| Right Postcentral Gyrus, Right Cuneal Cortex (7.0678e-07) | | | |
| Right Thalamus, Left Lateral Ventrical (7.2891e-07) | | | |

Reward sensitivity task significant connections for PTSD prediction at T3

*Format: region A, region B (p-value); sorted by significance.*

| Our Model | Ours E is the identity | Deep fMRI | Ours + GCN |
|---|---|---|---|
| Right Parahippocampal Gyrus, 'Left Frontal Medial Cortex (3.8601e-12) | None | None | Right Middle Temporal Gyrus, 'Left Amygdala' (4.383e-09) |
| Left Temporal Fusiform Cortex, , 'Left Middle Temporal Gyrus, (1.6451e-11) | | | Right Subcallosal Cortex', 'Left Paracingulate Gyrus (7.886e-08) |
| Left Frontal Orbital Cortex', 'Left Precentral Gyrus (2.779e-11) | | | Right Middle Temporal Gyrus, 'Brain-Stem (1.3752e-07) |
| Left Amygdala', 'Right Paracingulate Gyrus (6.383e-11) | | | Right Middle Temporal Gyrus, , 'Left Angular Gyrus (2.25982e-07) |
| Right Occipital Fusiform Gyrus', 'Right Planum Polare (6.844e-11) | | | Right Subcallosal Cortex', 'Right Cingulate Gyrus, (4.3198e-07) |
| Left Parahippocampal Gyrus, , 'Left Inferior Temporal Gyrus, (2.7531e-10) | | | Right Intracalcarine Cortex, Left Amygdala (4.8913e-07) |
| Left Inferior Frontal Gyrus, Right Parahippocampal Gyrus (4.074e-10) | | | Right Middle Temporal Gyrus,Left Parahippocampal Gyrus (6.384e-07) |
| Left Caudate', 'Right Parahippocampal Gyrus, anterior division (9.515e-10) | | | Right Middle Temporal Gyrus, , 'Right Planum Polare (8.8378e-07) |
| Left Hippocampus', 'Right Putamen (1.2084e-09) | | | Right Subcallosal Cortex', 'Left Superior Temporal Gyrus (8.8807e-07) |
| Right Frontal Medial Cortex', 'Right Parahippocampal Gyrus (1.0321e-08) | | | Right Middle Temporal Gyrus, Left Middle Temporal Gyrus, (9.175e-07) |
| Left Putamen', 'Right Juxtapositional Lobule Cortex (2.1504e-08) | | | Right Middle Temporal Gyrus, 'Right Supracalcarine Cortex') 9.572e-07 |
| Right Subcallosal Cortex', 'Left Inferior Temporal Gyrus, (2.5937e-08) | | | (Right Juxtapositional Lobule Cortex, 'Left Amygdala') 1.04358e-06 |
| Right Hippocampus', 'Right Parietal Operculum Cortex (2.7130e-08) | | | Right Superior Temporal Gyrus, 'Right Planum Polare') 1.1392e-06 |
| Left Cerebral White Matter', 'Right Putamen (2.7281e-08) | | | Left Supramarginal Gyrus, 'Left Middle Temporal Gyrus, 1.14615e-06 |
| Left Hippocampus', 'Right Cerebral White Matter (3.8891232e-08) | | | Right Supramarginal Gyrus, , 'Left Amygdala') 1.37441e-06 |
| Left Middle Temporal Gyrus, 'Right Frontal Medial Cortex (1.42974e-07) | | | Right Parahippocampal Gyrus, 'Left Paracingulate Gyrus') 1.43730e-06 |
| Left Hippocampus', 'Right Precentral Gyrus (2.35539e-07) | | | 'Left Temporal Fusiform Cortex, , 'Right Planum Polare') 1.48839e-06 |
| Right Planum Temporale', 'Left Thalamus (2.3945e-07) | | | Left Temporal Fusiform Cortex, 'Right Cingulate Gyrus 1.5299e-06 |
| Right Cerebral White Matter', 'Right Juxtapositional Lobule Cortex (3.0292e-07) | | | Right Middle Temporal Gyrus, 'Left Frontal Pole') 1.6373e-06 |
| Right Insular Cortex', 'Right Angular Gyrus (4.23892e-07) | | | Right Subcallosal Cortex', 'Left Cuneal Cortex') 1.7276e-06 |
| Right Cerebral White Matter', 'Right Lateral Occipital Cortex(5.12025e-07) | | | Right Frontal Operculum Cortex', 'Left Amygdala') 1.735e-06 |
| Left Amygdala', 'Right Supramarginal Gyrus, anterior division (6.59903e-07) | | | Right Inferior Frontal Gyrus,'Left Amygdala') 1.74073e-06 |
| Right Subcallosal Cortex', 'Right Temporal Fusiform Cortex, (7.87381e-07) | | | Right Superior Temporal Gyrus, 'Brain-Stem') 1.79771e-06 |
| Left Temporal Fusiform Cortex, anterior division', 'Left Temporal Pole (8.42961e-07) | | | Right Middle Temporal Gyrus, 'Right Parahippocampal Gyrus, 1.932e-06 |
| Right Planum Temporale', 'Left Temporal Occipital Fusiform Cortex (1.04586e-06) | | | Left Parahippocampal Gyrus, , 'Left Amygdala') 3.07474e-06 |
| Left Hippocampus', 'Right Inferior Temporal Gyrus, posterior division (1.492977e-06) | | | Right Middle Temporal Gyrus, 'Left Cerebral White Matter3.384e-06 |
| Left Hippocampus', 'Right Pallidum (2.2293e-06) | | | |
| Right Cingulate Gyrus, , 'Right Juxtapositional Lobule Cortex (3.0161e-06) | | | |
| Right Planum Temporale', 'Right Hippocampus (3.2904e-06) | | | |
| Left Amygdala', 'Left Frontal Pole (4.108e-06) | | | |
| Right Planum Temporale', 'Left Supramarginal Gyrus, anterior division (4.6602e-06) | | | |
| Right Planum Temporale', 'Left Hippocampus (4.9401e-06) | | | |
| Left Temporal Fusiform Cortex, posterior division', 'Right Cuneal Cortex (6.1653e-06) | | | |
| Right Putamen', 'Brain-Stem (6.76948e-06) | | | |
| Left Middle Temporal Gyrus,'Left Parahippocampal Gyrus, (7.7971e-06) | | | |
| Left Amygdala', 'Left Cerebral Cortex (1.0052e-05) | | | |
| Left Superior Frontal Gyrus', 'Right Angular Gyrus (1.1906e-05) | | | |
| Left Temporal Fusiform Cortex, , 'Left Paracingulate Gyrus (1.2768e-05) | | | |
| Right Lateral Occipital Cortex, , 'Left Middle Temporal Gyrus, (1.2814e-05) | | | |
| Left Cerebral White Matter', 'Brain-Stem (1.3415e-05) | | | |
| Right Planum Temporale', 'Left Parietal Operculum Cortex (1.4677e-05) | | | |
| Left Hippocampus', 'Left Parahippocampal Gyrus, anterior division (1.5243e-05) | | | |
| Left Hippocampus', 'Brain-Stem (1.8140e-05) | | | |
| Right Cerebral White Matter', 'Left Putamen (2.2339e-05) | | | |
| Right Subcallosal Cortex', 'Left Cingulate Gyrus, anterior division (2.5178e-05) | | | |
| Left Temporal Fusiform Cortex, 'Left Inferior Frontal Gyrus, (2.73640e-05) | | | |
| Right Central Opercular Cortex', 'Right Subcallosal Cortex (2.7431e-05) | | | |
| Right Central Opercular Cortex', 'Left Parahippocampal Gyrus, (2.8318e-05) | | | |
| Left Insular Cortex', 'Right Superior Temporal Gyrus, anterior division (3.1294e-05) | | | |
| Left Cingulate Gyrus, anterior division', 'Left Temporal Fusiform Cortex, (3.3139e-05) | | | |
| Left Cingulate Gyrus, , 'Left Temporal Occipital Fusiform Cortex (3.6362e-05) | | | |
| Left Inferior Temporal Gyrus, , 'Left Frontal Medial Cortex (3.8287e-05) | | | |
| Left Frontal Orbital Cortex', 'Right Temporal Fusiform Cortex, (4.229e-05) | | | |
| Right Planum Temporale', 'Right Supramarginal Gyrus, (4.4743e-05) | | | |
| Right Planum Temporale', 'Right Central Opercular Cortex (5.0616e-05) | | | |
| Left Temporal Fusiform Cortex, anterior division', 'Right Putamen (5.0889e-05) | | | |
| Left Temporal Fusiform Cortex, anterior division', 'Right Amygdala (5.9604e-05) | | | |
| Left Insular Cortex', 'Right Inferior Frontal Gyrus, pars opercularis (6.5992e-05) | | | |
| Left Temporal Fusiform Cortex, anterior division', 'Right Temporal Pole (7.3566e-05) | | | |
| Left Occipital Fusiform Gyrus', 'Left Parietal Operculum Cortex (8.5796e-05) | | | |
| Right Parahippocampal Gyrus, posterior division', 'Left Temporal Pole (9.1234e-05) | | | |
| Left Paracingulate Gyrus', 'Left Temporal Occipital Fusiform Cortex (9.5061e-05) | | | |
| Right Lateral Occipital Cortex, inferior division', 'Right Pallidum (9.6424e-05) | | | |
| Left Insular Cortex', 'Left Lateral Occipital Cortex, inferior division (0.0001) | | | |
| Right Middle Temporal Gyrus, 'Left Inferior Frontal Gyrus, pars opercularis (0.0001) | | | |
| Left Insular Cortex', 'Right Inferior Frontal Gyrus, pars triangularis (0.0001) | | | |
| Left Hippocampus', 'Left Cerebral White Matter (0.00010254331698065488 | | | |
| Right Insular Cortex', 'Left Cingulate Gyrus, posterior division (0.0001) | | | |

| | | | |
|---|---|---|---|
| Left Juxtapositional Lobule Cortex, 'Right Lateral Ventricle (0.0001) | | | |
| Right Planum Temporale', 'Left Superior Temporal Gyrus, posterior division (0.0001) | | | |
| Left Middle Temporal Gyrus, , 'Left Inferior Frontal Gyrus, pars triangularis (0.0001) | | | |
| Left Temporal Pole', 'Left Cingulate Gyrus, anterior division (0.0001) | | | |
| Right Parietal Operculum Cortex', 'Right Occipital Fusiform Gyrus (0.0001) | | | |
| Right Parahippocampal Gyrus, 'Left Middle Temporal Gyrus(0.00014) | | | |
| Left Insular Cortex', 'Right Occipital Fusiform Gyrus (0.0001) | | | |
| Right Planum Temporale', 'Right Inferior Frontal Gyrus, pars triangularis (0.0001) | | | |
| Left Frontal Medial Cortex', 'Right Lateral Occipital Cortex, inferior division (0.0001) | | | |
| Left Inferior Temporal Gyrus, posterior division', 'Left Cuneal Cortex (0.0001) | | | |
| Right Planum Temporale', 'Left Superior Frontal Gyrus (0.0001) | | | |
| Right Planum Temporale', 'Left Inferior Frontal Gyrus, pars triangularis (0.0001) | | | |
| Left Parahippocampal Gyrus, 'Left Temporal Fusiform Cortex(0.0001) | | | |
| Right Lateral Occipital Cortex, inferior division', 'Brain-Stem (0.0001) | | | |
| Right Planum Temporale', 'Left Frontal Operculum Cortex (0.0001) | | | |
| Brain-Stem', 'Right Putamen (0.0001) | | | |
| Right Putamen', 'Left Cerebral White Matter (0.0001) | | | |
| Right Juxtapositional Lobule Cortex , 'Right Amygdala (0.0001) | | | |
| Right Central Opercular Cortex', 'Left Occipital Pole (0.0002) | | | |
| Right Juxtapositional Lobule Cortex (formerly Supplementary Motor Cortex)', 'Left Inferior Temporal Gyrus, posterior division (0.0002) | | | |
| Right Parahippocampal Gyrus, , 'Left Inferior Frontal Gyrus, pars triangularis (0.0002) | | | |
| Right Planum Temporale', 'Left Pallidum (0.0002) | | | |
| Left Middle Temporal Gyrus, temporooccipital part', 'Right Cuneal Cortex (0.0002) | | | |
| Right Insular Cortex', 'Right Inferior Frontal Gyrus, pars opercularis (0.0002) | | | |
| Right Subcallosal Cortex', 'Left Temporal Fusiform Cortex, posterior division (0.0002) | | | |
| Right Insular Cortex', 'Right Occipital Pole (0.0002) | | | |
| Left Frontal Medial Cortex', 'Left Subcallosal Cortex (0.0002) | | | |
| Right Lateral Ventricle', 'Right Inferior Frontal Gyrus, pars triangularis (0.0002) | | | |
| Left Insular Cortex', 'Right Superior Frontal Gyrus (0.0002) | | | |
| Right Lateral Ventricle', 'Left Temporal Occipital Fusiform Cortex (0.0002) | | | |
| Right Superior Temporal Gyrus, posterior division', 'Right Angular Gyrus (0.0002) | | | |
| Right Subcallosal Cortex', 'Left Frontal Orbital Cortex (0.0002) | | | |
| Left Frontal Orbital Cortex', 'Right Temporal Pole (0.0002) | | | |
| Right Cerebral White Matter', 'Right Parahippocampal Gyrus, (0.0002) | | | |
| Right Lateral Occipital Cortex, 'Left Inferior Temporal Gyrus, (0.0003) | | | |
| Right Central Opercular Cortex', 'Left Inferior Frontal Gyrus, pars opercularis (0.0003) | | | |
| Left Temporal Fusiform Cortex, 'Right Parahippocampal Gyrus, (0.0003) | | | |
| Right Planum Temporale', 'Right Temporal Pole (0.0003) | | | |
| Right Amygdala', 'Left Temporal Fusiform Cortex, anterior division (0.0003) | | | |
| Left Frontal Medial Cortex', 'Left Temporal Pole (0.0003) | | | |
| Right Middle Temporal Gyrus, posterior division', 'Left Inferior Frontal Gyrus(0.0003) | | | |
| Right Inferior Frontal Gyrus, pars triangularis', 'Left Accumbens (0.0003) | | | |
| Left Pallidum', 'Right Lateral Occipital Cortex, inferior division (0.0003) | | | |
| Right Planum Temporale', 'Right Temporal Occipital Fusiform Cortex (0.0003) | | | |
| Right Planum Temporale', 'Left Precentral Gyrus (0.0004) | | | |
| Left Hippocampus', 'Left Putamen (0.0004) | | | |
